# Global warming pushes the distribution range of the two alpine ‘glasshouse’ *Rheum* species north- and upwards in the Eastern Himalayas and the Hengduan Mountains

**DOI:** 10.3389/fpls.2022.925296

**Published:** 2022-10-07

**Authors:** Santosh Kumar Rana, Hum Kala Rana, Jürg Stöcklin, Sailesh Ranjitkar, Hang Sun, Bo Song

**Affiliations:** ^1^ Key Laboratory for Plant Diversity and Biogeography of East Asia, Kunming Institute of Botany, Chinese Academy of Sciences, Kunming, China; ^2^ Department of Ecosystem Science and Management, Pennsylvania State University, University Park, PA, United States; ^3^ Institute of Botany, University of Basel, Basel, Switzerland; ^4^ N. Gene Solution of Natural Innovation, Kathmandu, Nepal; ^5^ School of Development Studies, Lumbini Buddhist University, Devdaha, Nepal; ^6^ MICD, Faculty of Humanities and Social Science, Mid-West University, Lalitpur, Nepal; ^7^ Yunnan Key Laboratory for Integrative Conservation of Plant Species with Extremely Small Populations, Kunming Institute of Botany, Chinese Academy of Sciences, Kunming, China

**Keywords:** biodiversity hotspots, biomod2, climate change, glasshouse, range shifts, *Rheum*

## Abstract

Alpine plants’ distribution is being pushed higher towards mountaintops due to global warming, finally diminishing their range and thereby increasing the risk of extinction. Plants with specialized ‘glasshouse’ structures have adapted well to harsh alpine environments, notably to the extremely low temperatures, which makes them vulnerable to global warming. However, their response to global warming is quite unexplored. Therefore, by compiling occurrences and several environmental strata, we utilized multiple ensemble species distribution modeling (eSDM) to estimate the historical, present-day, and future distribution of two alpine ‘glasshouse’ species *Rheum nobile* Hook. f. & Thomson and *R. alexandrae* Batalin. *Rheum nobile* was predicted to extend its distribution from the Eastern Himalaya (EH) to the Hengduan Mountains (HM), whereas *R. alexandrae* was restricted exclusively in the HM. Both species witnessed a northward expansion of suitable habitats followed by a southerly retreat in the HM region. Our findings reveal that both species have a considerable range shift under different climate change scenarios, mainly triggered by precipitation rather than temperature. The model predicted northward and upward migration for both species since the last glacial period which is mainly due to expected future climate change scenarios. Further, the observed niche overlap between the two species presented that they are more divergent depending on their habitat, except for certain regions in the HM. However, relocating appropriate habitats to the north and high elevation may not ensure the species’ survival, as it needs to adapt to the extreme climatic circumstances in alpine habitats. Therefore, we advocate for more conservation efforts in these biodiversity hotspots.

## Introduction

“*Unless we move quickly to protect global biodiversity, we will soon lose most of the species composing life on Earth.*” —Edward O. Wilson (1929–2021).

Global biodiversity hotspots (Myers et al., 2000) harbor high levels of species richness and endemism (Sun et al., 2017; Rahbek et al., 2019), yet they are vulnerable to large-scale climatic change (Baker and Moseley, 2007). Two biodiversity hotspots, i.e., the Himalayas and the Mountains of Southwest China (also known as the Hengduan Mountains) (Myers et al., 2000; Mittermeier et al., 2011) known to be the biodiversity reservoir of ‘the Third Pole’, can’t remain intangible by present-day global warming (Qiu, 2008). The alpine areas of this region are characterized by low temperatures, intense solar radiation, strong winds, dense cloudiness, frequent precipitation, and a short growing season (He et al., 2006). Several plant species in this area have evolved specific phenotypes, such as “downy plant”, “cushion plant”, and “glasshouse plant” (Tsukaya and Tsuge, 2001; Körner, 2003) . But those plants, which are well adapted to extreme environments through specialized structures, might be susceptible to climate warming (Doiron et al., 2014). Nevertheless, plant species are known to respond to changes in climate by altering their phenology (Cleland et al., 2007) or shifting their distribution range (Parmesan and Yohe, 2003; Chen et al., 2011). Meanwhile, in the past plant species in the Himalaya-Hengduan Mountains (HHM) have been transformed by quaternary climatic oscillations (Sandel et al., 2011; Muellner-Riehl et al., 2019).

Global warming pushes the plant populations to upslope along elevation and poleward along latitude to track isotherms (Lenoir and Svenning, 2013; Zu et al., 2021). As mountain species migrate upslope to cooler climatic conditions to avoid rising temperatures, it results in a smaller inhabited area and population reduction (Morueta-Holme et al., 2015; Freeman et al., 2018). As they ascend higher in elevation towards mountaintops, the elevational range becomes progressively constrained, perhaps increasing the risk of extinction (Manne et al., 1999; Freeman et al., 2018). Warming temperature drives lowlands species to move upwards, and there may be no record for species adapted to higher temperatures to compensate for the loss (Colwell et al., 2008). Indeed, lowland species from warmer microhabitats may relocate to cooler refuges at the same elevation due to global warming (Bush et al., 2004). However, alpine plants might face summit trap phenomena (Rana et al., 2017; Salick et al., 2019). Due to climate change, plant distribution patterns are changing, with species expanding in more suitable areas and declining in increasingly hostile ones (Kelly and Goulden, 2008). Notably, past temperature rise presented an unusual and persistent upward movement of tree lines for about 1000 m in the Himalayan alpine meadows (Schickhoff et al., 2014) or the HM coniferous forest (Lenoir et al., 2008; Wang et al., 2018; Yao et al., 2020). In line with these predictions, several alpine plants (for instance, *Anemone rivularis*, *Abies delavayi*) have shifted their ranges upward, north-westward (Liang et al., 2018), or upslope (Kelly and Goulden, 2008; Lenoir et al., 2008; Rumpf et al., 2018). However, on the other hand, the alpine glasshouse species are rarely explored for their range shifts.

The genus *Rheum* (Polygonaceae) has around 60 species widely distributed across the QTP’s high latitudes. Only a few species make it to Central and Western Asia and Europe (Losina-Losinskaya, 1936; Kao and Cheng, 1975). Owing to the therapeutic and pharmacological properties of *Rheum*, the increased market demand has impaired the survival and existence of wild species (Chen et al., 2018). The majority of *Rheum* species have evolved specific morphologies to cope with the harsh climate (Tsukaya and Tsuge, 2001). The ‘glasshouse’ plants, with their large upper translucent bracts covering the inflorescences, are examples of such adaptations (Ohba, 1988). Despite the fact ‘glasshouse’ morphology may be found in more than ten plant families, including Ranunculaceae, Caryophyllaceae, Lamiaceae, Asteraceae, and Polygonaceae (Yoshida, 2002), the most known alpine ‘glasshouse’ plant species that have piqued the interest of evolutionary and conservation biologists are *R. nobile* Hook. f. & Thomson and *R. alexandrae* Batalin. The multifunctional translucent bracts of such plants are likely to have evolved independently in the Himalayas as a response to low temperatures and high irradiance, facilitating an upward range shift in response to climate change (Tsukaya and Tsuge, 2001; Tsukaya, 2002; Sun et al., 2012; Song et al., 2013c; Song et al., 2015; Song et al., 2020a). On the other hand, cold-adapted alpine plants may experience range contractions and/or local extinctions (Giménez-Benavides et al., 2011; Wiens, 2016) due to a slower rate of adaptation than that of climate change (Quintero and Wiens, 2013). Therefore, to fill the unprecedented research gap in *Rheum* species and to develop a scientific strategy to protect alpine glasshouse species (i.e., *R. nobile* and *R. alexandrae*) from the negative consequences of global warming, we used species distribution models and assessed the geographical distribution range shift.

The species distribution models (SDM) combined with geographic information systems have ushered in a new era of research into the consequences of climate change on species ecology, biogeography, and conservation (Guisan and Thuiller, 2005; Warren et al., 2008). Ensemble SDM (eSDM) can discover geographic places with a high possibility of having a focal species present by focusing on biological niche demands (Guisan and Thuiller, 2005). With the recent advancement in SDM approaches (Miller, 2010), multi-model projections have been widely utilized to identify the climatic envelope and range shift for endemic and endangered species (Pearson et al., 2007; Robinson et al., 2018).

This research looks at how global warming affects the distribution ranges of the geographically co-occurring alpine ‘glasshouse’ species *R. nobile* and *R. alexandrae* (Polygonaceae). With the following precise goals in mind, we adopted multiple eSDM approaches to 1) characterize the realized niche underpinning the distribution of the two focal species, 2) predict range shifts of the two focal species under various climatic scenarios, and 3) discuss potential adaptive responses to changes in the distributional range of the two focal species in response to global warming for biodiversity conservation.

## Materials and methods

### Focal species

**Figure 1:**
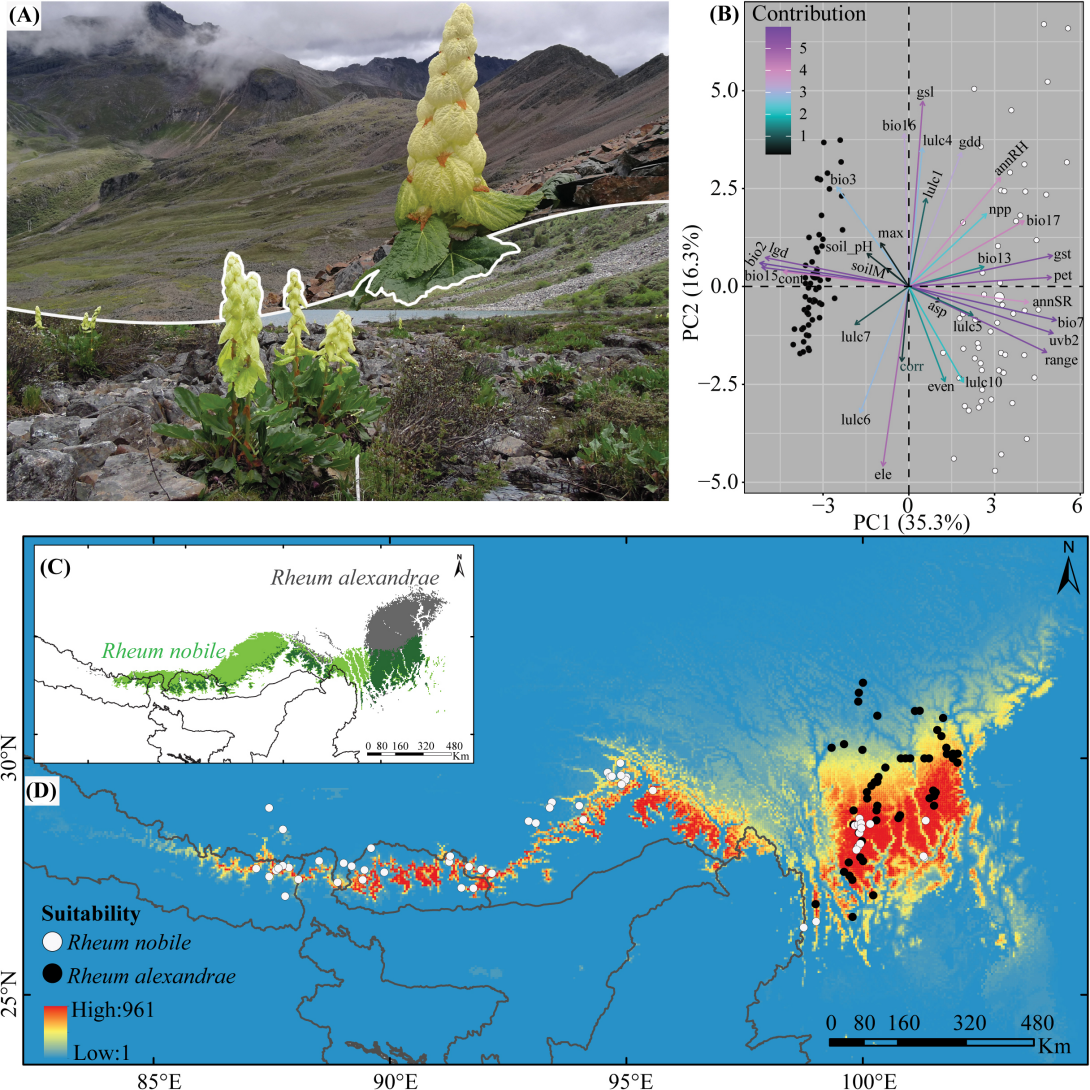
**(A)** Images of *Rheum nobile* (above) and *Rheum alexandrae* (below) in their respective habitats. **(B)** The relative contribution of the predictive environmental variables (colored arrows) to the two axes of biplots from principal components analysis (PCA). The distribution area of both species as a function of **(C)** raster overlay and **(D)** fuzzy overlay depicts their present-day bio-climatically suitable niche in the Eastern Himalaya (EH) and the Hengduan Mountains (HM). The two dots in **(B, D)** represent the occurrence points for *R. nobile* (white dot) and *R. alexandrae* (black dot).

*Rheum nobile* and *R. alexandrae* are two species that have evolved specific adaptations to cope with the cold-foggy and damp conditions prevalent at high elevations in the HHM, respectively. Both species (**Figure 1A**) are giant perennial herbs endemic to the Eastern Himalayas (EH) and the Hengduan Mountains (HM) (**Figure 1D**), growing at elevations between 3,400 and 6,000 m a.s.l. (Li and Gao, 1998; Chowdhery and Agrawala, 2009). *Rheum nobile* is a monocarpic herb with individuals dying after a single reproductive event after ca. 33 years of vegetative growth (Song et al., 2020b), whereas *R. alexandrae* is a multi-stemmed polycarpic perennial (Song et al. unpublished). Despite their relatedness and morphological similarity (Sun et al., 2012), they have distinct habitat and range distributions. *Rheum nobile* is found chiefly on alpine open scree and occasionally in open patches of alpine meadows, i.e., in well-drained habitats (Chowdhery and Agrawala, 2009; Song et al., 2013c), whereas the partly sympatric *R. alexandrae* is found mainly on alpine wetlands, including marshes, swampy meadows, and lakeshores (Chen, 1993).

### Rarefaction of species occurrence

We focused on the entire geographic distribution ranges of the two species (**Figure 1D**). For this, the past field (1937–2020) (**Supplementary Table S1**) datasets were compiled and cross-checked with the herbarium records from the National Herbarium and Plant Laboratories (KATH, Nepal) and Kunming Institute of Botany, CAS (KUN, China) to construct the geographic distribution of both species. In addition to past field and herbarium collections, we also ground-validated occurrence points (from 2015 to 2022) through GPS in the field and cross-checked with the online databases of the Chinese National Herbarium (PE; http://pe.ibcas.ac.cn/en/), Chinese Virtual Herbarium (CVH; http://www.cvh.ac.cn/), the Global Biodiversity Information Facility (GBIF.org, 2021), the Royal Botanical Garden at Edinburgh (RBGE, United Kingdom; http://data.rbge.org.uk/search/herbarium/) and the Herbarium at the University of Tokyo (TI, Japan; http://umdb.um.u-tokyo.ac.jp/DShokubu/). While compiling the occurrence datasets, we mainly focused on geographic coordinates recorded by the different resource persons during the field. So, the occurrence points from the other herbarium specimens and online databases were discarded in the datasets. Following Barbet-Massin et al. (2012), the background points were randomly chosen within a 25–50 km radius of the occurrence points.

Overfitting or biases in eSDM may result from geo-coding errors in the herbarium label and spatially clustered localities (Hijmans, 2012; Boria et al., 2014; Rana et al., 2021), making it challenging to forecast range distribution and climatic suitability accurately. Therefore, we spatially rarefied the gathered geographic occurrences to avoid model bias (Boria et al., 2014; Rana et al., 2017; Rana et al., 2020b). Due to the filter distances’ effectiveness in spatially high heterogeneous regions, like mountains, the occurrences were rarefied using a 10-km spatial grid (Veloz, 2009; Boria et al., 2014; Rana et al., 2021). Finally, the models were developed using 56 out of 79 *R. nobile* occurrences and 54 out of 77 *R. alexandrae* occurrences after rarefaction.

### Explanatory environmental variables

The potential distribution of the two focal species was modeled for the present-day (ca. 1990–2000) and projected to the paleo (LIG: last inter-glacial ca. 120,000–140,000 years BP; the LGM: last glacial maximum ca. 22,000 years BP) and future (2070, Representative Concentration Pathways, RCPs4.5) climatic scenarios. The climatic projection was created using a multi-model median (MMM) ensemble of General Circulation Models (GCMs) (Rana et al., 2021) (see **Supplementary Table S2** for GCMs).

We used three different eSDM in different environmental strata, each with its unique set of predictive variables. Firstly, bioclimatic variables (http://www.worldclim.org/version2) were used to forecast under paleo climatic (LIG, LGM), present-day, and future scenarios. Secondly, all environmental variables were pooled and forecasted. Finally, ensemble forecasting was performed by combining bioclimatic variables with other environmental variable categories such as geo-climatic (http://www.worldclim.org/version2; http://nelson.wisc.edu/sage; http://www.cgiar-csi.org), habitat heterogeneity (Tuanmu and Jetz, 2015; http://www.earthenv.org/texture), growing days (https://chelsa-climate.org/), ultra-violet radiation (Beckmann et al., 2014; https://www.ufz.de/gluv */*) and consensus land-cover (Tuanmu and Jetz, 2014; http://www.earthenv.org/landcover). All these climatic variables (Hijmans et al., 2005) and other environmental variables were used with a spatial resolution of 2.5 arc-min (**Table 1**).

**Table 1:** Multi-step selection of predictive environmental variables for ensemble species distribution modeling (eSDM) of two alpine ‘glasshouse’ herbs, *Rheum nobile* and *Rheum alexandrae*.

Environmental variables	*Rheum nobile*				*Rheum alexandrae*			
	1^st^	2^nd^	3^rd^		1^st^	2^nd^	3^rd^	
**Bioclimatic variables (*V1*)**								
Mean diurnal range (°C)	–	–			bio2	bio2		
Isothermality [(bio2/bio7) × 100]	bio3	bio3			bio3	bio3		
Temperature Annual Range (bio5-bio6) (°C)	bio7	bio7			–	–		
Mean Temperature of Wettest Quarter (°C)	–	–			bio8	–		
Mean Temperature of Warmest Quarter (°C)	bio10	–			–	–		
Precipitation of Wettest Month (mm)	bio13	bio13			bio13	bio13		
Precipitation Seasonality (Coefficient of Variation)	bio15	–			bio15	bio15		
Precipitation of Wettest Quarter (mm)	bio16	bio16			bio16	bio16		
Precipitation of Driest Quarter (mm)	bio17	bio17			bio17	–		
Precipitation of Coldest Quarter (mm)	bio19	–			–	–		
**Geo-climatic (*V2*)**								
Elevation (m)	ele	ele	ele	bio3, bio7 bio10, bio13 bio16, bio17 bio19	ele	ele	ele	bio2, bio3 bio8, bio13 bio15, bio16
Aspect (degree)	asp	asp	asp		asp	asp	asp	
Net primary productivity (kg-carbon/m²/year)	npp	npp	npp		npp	npp	npp	
Annual aridity (ratio)	ai	–	–		ai	–	–	
Soil moisture (mm based on 150 mm water holding capacity)	soilM	soilM	soilM		soilM	soilM	soilM	
Potential evapotranspiration (mm)	pet	pet	pet		pet	–	pet	
Annual Relative Humidity (%)	annRH	annRH	annRH		annRH	annRH	annRH	
Annual solar radiation (kJ/m²/day)	annSR	annSR	annSR		annSR	annSR	annSR	
Soil pH (acidity-alkaline level)	soil_pH	soil_pH	soil_pH		soil_pH	soil_pH	soil_pH	
Soil carbon (Kg-Carbon/m² to 1 m depth)	soilC	–	soilC		soilC	–	–	
Annual water vapour (kPa)	–	–	–		annWV	–	–	
**Habitat heterogeneity (*V3*)**								
Correlation (linear dependency of EVI on adjacent pixels, 1≥ x ≥-1)	corr	corr	corr	bio3, bio7 bio10, bio13 bio15, bio16 bio17, bio19	corr	corr	corr	bio2, bio3 bio8, bio13 bio15, bio16 bio17
Coefficient of variation (normalized dispersion of EVI, x ≥0)	cv	–	cv		cv	–	cv	
Evenness (evenness of EVI, 1≥ x ≥0)	even	even	even		even	even	even	
Homogeneity (similarity of EVI between adjacent pixels, 1≥ x ≥0)	homo	–	homo		homo	–	homo	
Maximum (dominance of EVI combinations between adjacent pixels, 1≥ x ≥0)	max	max	max		max	max	max	
Range (Range of EVI, x ≥0)	range	range	range		–	–	–	
Contrast (exponentially weighted difference in EVI between adjacent pixels, x ≥0)	–	–	–		cont	cont	cont	
**Growing days (*V4*)**								
Growing degree days (°C based on 5-degree base temperature)	gdd	gdd	gdd	bio3, bio7 bio13, bio15 bio16, bio17 bio19	gdd	gdd	gdd	bio2, bio3 bio8, bio13 bio15, bio16 bio17
Growing season length (number of days)	gsl	gsl	gsl		gsl	gsl	gsl	
Growing season temperature (°C/10)	gst	gst	gst		gst	–	–	
Last day of growing season (Julian day)	–	–	–		lgd	lgd	lgd	
**Ultra-violet radiations (*V5*)**								
UV-B seasonality (J/m²/day)	uvb2	uvb2	uvb2	bio3, bio7 bio10, bio13 bio15, bio16 bio17, bio19	uvb2	uvb2	uvb2	bio2, bio3 bio8, bio13 bio15, bio16 bio17
Mean UV-B of lowest month (J/m²/day)	uvb4	–	uvb4		–	–	–	
Sum of monthly mean UV-B during lowest quarter	uvb6	–	–		uvb6	–	–	
**Consensus landcover (*V6*)**								
Evergreen/deciduous Needleleaf trees (%)	lulc1	lulc1	lulc1	bio3, bio7 bio10, bio13 bio15, bio16 bio17, bio19	lulc1	lulc1	lulc1	bio2, bio3 bio8, bio13 bio15, bio16 bio17
Mixed/other trees (%)	lulc4	lulc4	lulc4		lulc4	lulc4	lulc4	
Shrubs (%)	lulc5	lulc5	lulc5		–	–	–	
Herbaceous vegetation (%)	lulc6	lulc6	lulc6		lulc6	lulc6	lulc6	
Cultivated and managed vegetation (%)	lulc7	lulc7	lulc7		lulc7	lulc7	lulc7	
Snow/ice (%)	lulc10	lulc10	lulc10					

1^st^, categories-wise selection; 2^nd^, all categories combinedly (for combined eSDM); 3^rd^, Bioclimatic variables with other environmental variables categories.

The elimination of highly correlated and/or redundant variables reduces the high collinearity, uncertainties, and predictive errors associated with clustered variables (Ranjitkar et al., 2014). Therefore, we used successive steps of variable selection. Firstly, variables were selected category-wise; secondly, all variables from each category were pooled together (for combined ensemble forecasting); and finally, bioclimatic variables were combined with environmental variables from other categories. Initially screened global consensus landcover variables were analyzed with species occurrences larger than a 30% threshold out of all occurrences (**Supplementary Table S3**). We used the variance inflation factor (VIF; Fox and Weisberg, 2011) (calculated using the R-package ‘car’ fixing elevation as a response variable) in conjunction with a Pearson correlation matrix as the significant core of variable selection. In VIF, the linear function of the model employs numeric response variables and is expressed as VIF*j*=1/(1–*R^2^j*), where *R^2^j* is the *R* ^2^-value obtained by regressing the *j* ^th^ predictor on the remaining predictors (Jackson et al., 2009). Variables with VIF*j* >10 indicate strong multi-collinearity (Quinn and Keough, 2002), and variables with a high VIF*j* have a negative impact on the modeling output. Several tests were run with extracted values of occurrences for bioclimatic and other environmental variables until a set of predictors with VIF*j* values less than 10 was retained. The selection of explanatory variables includes Pearson correlation values of < |0.8| (see **Supplementary Tables S4**, **S5** for correlation analysis results) and VIF*j* values of < 10 (see **Supplementary Tables S6**–**S10** for VIF*j* analysis results). Initially, variables were selected based on the Pearson correlation (**Supplementary Tables S4**, **S5**) and VIF*j* analysis (**Supplementary Tables S6**, **S7**). In contrast, the remaining steps used VIF (**Supplementary Tables S8**–**S10**) as the primary function of the variable selection for both focal species. Several species-specific and environmental variables were used as a subset of predictive variables for forecasting the suitable range (**Table 1**).

Furthermore, principal component analysis (PCA) was performed to group the occurrences according to the predictive bioclimatic and environmental variables of *R. nobile* and *R. alexandrae* using the R-package ‘*factoextra*’ (Kassambara and Mundt, 2020).

### Ensemble model development and validation

For a given species distribution model, we used the standardized approach in ODMAP (Overview, Data, Model, Assessment and Prediction) (Zurell et al., 2020) protocol structure (**Supplementary Methods**). The ensemble of SDMs was implemented using the R-package ‘*Biomod2’* (Thuiller et al., 2020). *Biomod2* outperforms single algorithms by incorporating simulations across multi-model classes, parameters, and climatic conditions (Araujo and New, 2007). Moreover, the consistent modeling approach was applied throughout the different strategies implemented for *R. nobile* and *R. alexandrae*. The assessment of SDM using niche-based modeling techniques allows for different modeling approaches such as bioclimatic envelopes, regression, classification methods, and machine learning methods (Thuiller et al., 2020) (**Table 2**). Except for MaxEnt with a maximum iteration of 5000 (Maximum Entropy Models; Phillips et al., 2006), all other models in *Biomod2* were run with the default settings.

**Table 2:** Model evaluation indices for the ensemble species distribution modeling (eSDM) of *Rheum nobile* and *Rheum alexandrae* using Biomod2 in R-programming language.

R. nobile/R. alexandrae	V1			V1 + V2			V1+ V3			V1+V4			V1+ V5			V1+ V6			Environmental		
***Algorithms***	*Kappa*	*TSS*	*AUC*	*Kappa*	*TSS*	*AUC*	*Kappa*	*TSS*	*AUC*	*Kappa*	*TSS*	*AUC*	*Kappa*	*TSS*	*AUC*	*Kappa*	*TSS*	*AUC*	*Kappa*	*TSS*	*AUC*
Generalized additive model (GAM)	0.92/1	0.98/1	1/1	1/1	1/1	1/1	1/1	1/1	1/1	1/1	1/1	1/1	0.95/1	0.99/1	1/1	1/1	1/1	1/1	1/1	1/1	1/1
Generalized boosting model (GBM)	0.96/0.97	1/1	1/1	0.99/0.97	1/1	1/1	0.97/0.99	1/1	1/1	0.97/0.96	1/1	1/1	0.97/0.98	1/1	1/1	0.96/0.98	1/1	1/1	0.98/0.99	1/1	1/1
Generalized linear model (GLM)	0.84/0.75	0.94/0.91	1/0.99	0.89/0.94	0.97/0.99	1/1	0.85/0.83	0.93/0.94	1/0.99	0.89/0.92	0.95/0.95	1/1	0.86/0.82	0.93/0.94	1/1	0.88/0.83	0.94/0.92	1/0.99	0.91/1	0.99/1	1/1
Classification tree analysis (CTA)	0.91/0.93	0.93/0.94	0.97/0.99	0.91/0.93	0.91/0.99	0.97/1	0.88/0.91	0.85/0.94	0.96/0.97	0.9/0.88	0.89/0.85	0.96/0.93	0.9/0.92	0.91/0.92	0.97/0.99	0.91/0.91	0.93/0.94	0.97/0.97	0.9/0.88	0.85/0.85	0.94/0.93
Artificial neural network (ANN)	0.84/0.99	0.8/0.98	0.96/0.99	0.98/0.95	0.98/0.98	1/1	0.91/0.85	0.93/0.97	0.99/0.98	0.85/0.97	0.97/0.98	0.99/1	0.89/0.97	0.89/0.96	0.96/0.99	0.92/1	0.93/1	0.99/1	0.79/0.86	0.82/0.85	0.93/0.98
Flexible discriminant analysis (FDA)	0.87/0.89	0.93/0.98	0.98/1	0.84/0.87	0.89/0.99	0.95/1	0.87/0.86	0.89/0.98	0.96/1	0.9/0.87	0.97/0.94	1/0.99	0.85/0.88	0.94/0.97	0.98/1	0.86/0.9	0.91/0.98	0.96/1	0.92/0.94	0.97/0.98	0.99/0.99
Multiple adaptive regression splines (MARS)	0.9/0.95	0.97/0.98	1/1	1/0.91	1/0.89	1/0.94	0.9/0.96	0.97/0.98	1/0.99	0.94/0.94	0.97/0.99	1/1	0.9/0.95	0.94/0.98	0.99/1	0.92/0.98	0.95/1	1/1	1/1	1/1	1/1
Random forest (RF)	1/0.99	1/1	1/1	1/1	1/1	1/1	0.99/1	1/1	1/1	1/0.99	1/1	1/1	1/0.99	1/1	1/1	0.99/1	1/1	1/1	1/1	1/1	1/1
Surface range envelops (SRE)	0.7/0.82	0.62/0.72	0.81/0.86	0.65/0.69	0.52/0.56	0.76/0.78	0.6/0.67	0.48/0.52	0.74/0.76	0.7/0.81	0.6/0.7	0.8/0.85	0.67/0.81	0.55/0.7	0.78/0.85	0.61/0.77	0.48/0.65	0.74/0.82	0.46/0.62	0.32/0.46	0.66/0.73
Maximum Entrophy (MaxEnt)	0.93/0.96	0.98/0.97	1/0.99	0.99/0.95	1/0.98	1/1	0.95/0.99	0.99/1	1/1	0.95/0.95	0.97/0.98	1/1	0.92/0.95	0.97/0.98	1/1	0.97/0.97	0.97/0.98	1/0.99	1/1	0.9/1	1/1

Kappa, Cohen’s kappa; TSS, True Skill Statistics; AUC, Area Under the Curve; V1, Bioclimatic variables; V2, Geo-climatic; V3, Habitat-heterogeneity; V4, Growing days; V5, Ultra-violet radiations; V6, Consensus landcover

Apart from analyzing significant variables, the essential steps in modeling are ensemble model development and validation for consensus mapping, which is generally done using a four-step modeling procedure in *Biomod2*. The initial step was calibrating ten sub-models with the ‘*BIOMOD_Modelling*’ function, which defined 4-fold cross-validation using 75% of the data to train the models. The remaining 25% were used to assess the predictive power using True Skill Statistics (TSS), Cohen’s Kappa, and Area Under Curve-Receiver Operating characteristics (AUC) statistics (Araujo et al., 2005; Allouche et al., 2006). The threshold-dependent model accuracy matrices, i.e., TSS and Cohen’s kappa, are independent of prevalence–the ratio of presence to pseudo-absence data in presence-absence predictions (Allouche et al., 2006). It considers both sensitivity and specificity, with the values ranging from −1 to +1, with +1 denoting perfect agreement. Scores between 0.6 to 0.9 suggest medium to good model performance (Allouche et al., 2006). The threshold-independent model evaluation indicator AUC, on the other hand, is likewise independent of prevalence (Phillips et al., 2006) and examines the models’ discriminating abilities. AUC values of less than 0.6 were regarded as poor, 0.6–0.9 were rated moderate, and >0.9 were considered excellent (Phillips et al., 2006). We applied the ‘*BIOMOD_EnsembleModeling*’ function in the second step, with sub-model weights > 0.9 assessed by TSS for ensemble modeling. The sampling procedure was replicated five times. We then used ‘*BIOMOD_Projection*’ for projecting the calibrated sub-models into a new space or time. Finally, the ‘*BIOMOD_EnsembleForecasting*’ function was used to forecast and generate the consensus mapping of species over time and space. The consensus model was then projected onto the past (LIG, LGM) and future (2070) climatic scenarios. Each model’s contribution to the final ensemble model was proportional to its goodness-of-fit statistics.

### Mapping the ensemble species distribution model

The spatial conversion of the consensus ensemble model to a binary model (presence/absence) was based on the thresholds (50% of suitable habitats) that suit the present-day distribution of the focal species (Forester et al., 2013; Rana et al., 2020a; Rana et al., 2021). The spatial analyses were carried out in ArcMap 10.4.1 (Environmental Systems Resource Institute (ESRI), 2016) using the extension Spatial Analysis to reclassify changes in LIG, LGM, and future conditions compared to present-day suitability into reduction, stable, and expanded areas of the focal species. The predicted suitable habitat maps of the two focal species were then overlapped to locate overlapping regions under present-day climatic scenarios.

### Niche equivalency test

Under the current bioclimatic scenario, we used the *ecospat* test in R-package ‘*ENMTools*’ (Warren and Dinnage, 2021) for the two focal species, *R. nobile*, and *R. alexandrae*, to test the hypothesis that the environmental niche model formed by population occurrences is identical. The R version of *ENMTools* has a more straightforward user interface of *ecospat* hypothesis testing with *enmtools.species* objects. It further conducts principal component analysis for multiple predictor variables to reduce them to a two-dimensional environment space (Warren et al., 2021). The bioclimatic niches were measured and validated using Schoener’s ‘*D*’ and Hellinger’s-based ‘*I*’ through *enmtools.ecospat.id* function of identity test and *enmtools.ecospat.bg* function of background test (symmetric and asymmetric) (Warren et al., 2010; Warren et al., 2021). Schoener’s *D* is a formula that calculates the appropriate range depending on the probability of occupied grid cells. Hellinger’s-based *I* works in a similar way as Schoener’s *D*, but without the assumption (Warren and Seifert, 2011). The pairwise similarity values of *D* and *I* indices ranged from 0 (complete divergence/no overlap) to 1 (high similarity/complete overlap), indicating that as the score increases, so does the niche overlap.

## Results

### Explanatory variables analysis and model performance

**Figure 2:**
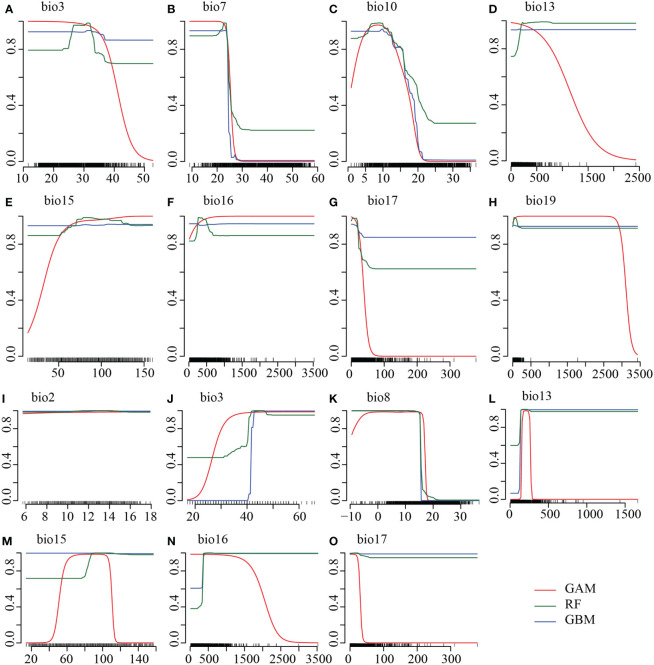
Response curves of the highly predictive top three algorithms showing the probability of presence (y-axis) under the **(A–C, I–K)** temperature-dependent and **(D–H, L–O)** precipitation-dependent variables (x-axis) for **(A–H)** *Rheum nobile* and **(I–O)** *Rheum alexandrae*. The predictive algorithms are the Generalised Additive Model (GAM; red line), Generalised Boosting Models (GBM; blue line), and Random Forest (RF; green line), with high model accuracy TSS > 0.9. Temperatures are expressed in °C (degree Celsius) and precipitation in mm (millimeter). Refer to **Table 1** for the bioclimatic variables.

The series of variable selection resulted in a robust set of bioclimatic and other environmental variables to predict the environmental niche of the two species. Initially screened variable selection through Pearson correlations (r <|0.8|) and VIF (VIF*j* <10) yielded 36 and 34 least correlated and non-redundant variables for *R. nobile* and *R. alexandrae*, respectively. These variables are species-specific to determine the distribution of *R. nobile* and *R. alexandrae* (refer to **Table 1** for details on predictive variables). Landcover, being an influential factor in determining species range for SDM (Bucklin et al., 2015), we chose the number of occurrences found within each landcover variable (**Supplementary Table S3**). The first model employs bioclimatic variables, including eight for *R. nobile* (three temperature-dependent, five precipitations dependent) and seven for *R. alexandrae* (three temperature-dependent, four precipitations-dependent). Highly contributing algorithm with TSS >0.9 (**Table 2**) were selected to estimate the response curves of the variables, notably for bioclimatic variables from eSDM (**Figure 2**) and *ENMTools* (see **Supplementary Figure S1**).

The suitability of *R. nobile* is more favored by temperature annual range (bio7: ca. 28 ˚C), mean temperature of warmest quarter (bio10: ca. 10 ˚C), precipitation of coldest quarter (bio19: ca. 12 mm) than that of *R. alexandrae* by mean diurnal range (bio2: ca. 14 ˚C), mean temperature of the wettest quarter (bio8: 8 ˚C) (**Figure 2** **;** **Supplementary Figure S1**). Furthermore, the co-existing niches for both focal species are characterized by isothermality (bio3; ca. 45), precipitation of wettest month (bio13, ca. 200 mm), precipitation seasonality (bio15; ca. 100 mm), precipitation of wettest quarter (bio16; 300–500 mm), and precipitation of driest quarter (bio17; ca. 12 mm) (**Figure 2** **;** **Supplementary Figure S1**). However, other environmental variables contributed to forecasting suitable habitats for the two focal species. When all predictive environmental variables were combined as in the second modeling, robust comparable model algorithms were produced (**Table 2**). Also, in the third modeling when all these environmental variables were independently modeled with bioclimatic variables only, suitability was found to be predictive.

Meanwhile, the first two components of a PCA with all predictive variables explained 51.6% (PC1: 35.3% and PC2: 16.3%) of the observed variance for the two focal species, and their environmental niches were demarcated from each other in biplots (**Figure 1B** **;** **Supplementary Figure S2**). Bioclimatically (*V1*), bio2, bio3, and bio15 are the most important for *R. alexandrae*, whereas bio7 and bio17 are the most important for *R. nobile*. Although the contributions of the different variables in the biplots are species-specific, *R. nobile*’s niche is characterized by an assembly of most variables. When compared to *R. alexandrae*, the niche of *R. nobile* is characterized largely by environmental variables (refer to **Figure 1B** and **Table 1** for more details on variables contribution). The model evaluation indices AUC and Cohen’s kappa value varied from 0.66 to 1 and 0.46 to 1. The SRE algorithm scored the lowest, and the RF, GAM, and GBM algorithms scored the highest (**Table 2**). Finally, the consensus model was evaluated and calibrated for all three modelings, with TSS >0.9 (details in **Table 2**). The result indicates that the consensus models were highly predictive and accurate regarding AUC, Cohen’s kappa, and TSS (**Table 2**).

### Ecological niche analysis

**Figure 3:**
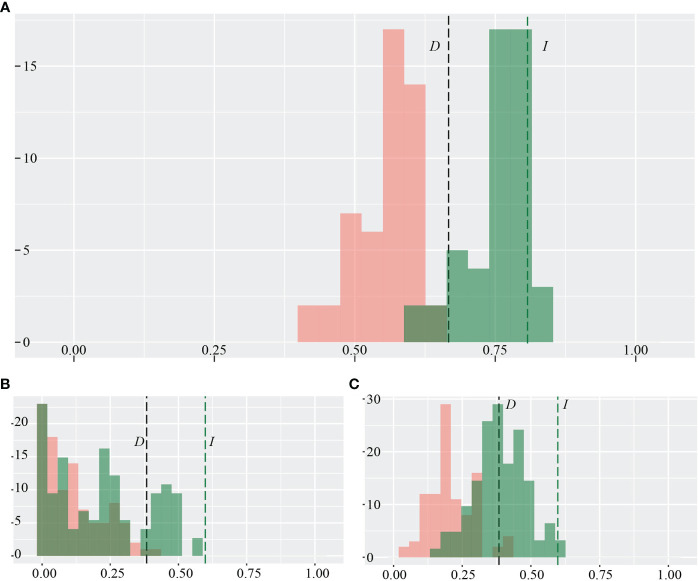
Pairwise niche **(A)** identity test and background test **(B)**, symmetric; **(C)**, asymmetric calculated between the two alpine ‘glasshouse’ herbs *Rheum nobile* and *Rheum alexandrae* as a function of *niche.overlap* ecospat test implemented in ENMtools. The niche equivalency test as species-wise pair comparisons was measured by Schoener’s *D* (in black color) and Hellinger’s-based *I* (in green color) indices.

Quantitatively, the average weight of the identity test (*D, I* < 0.82; **Figure 3A**) and the low weight of the background test (*D, I*<0.6; **Figures 3B, C**) show that the two focal species *R. nobile* and *R. alexandrae*, have quite different environmental niches. The ENM similarity score for the present-day occurrence of two species is average compared to what would be expected based on the null hypothesis of niche equivalency, indicating that the two species’ environmental niches may not be similar (**Figure 3A**). However, the observed overlap between the two species in background tests is lower than expected under the null hypothesis, showing that the two species are more divergent depending on their habitat (**Figures 3B, C**). However, the predictive power of the niche assemblage differs significantly as a consequence of multiple algorithms. The lowest to average score on the identity test and background test through GAM indicates that the two species are ecologically apart (**Supplementary Figures S3A, C, E**). In contrast, RF algorithms with the highest identity and background tests imply that the two species’ ecological niches are comparable and co-occur at the same locality (**Supplementary Figures S3B, D, F**).

### Climate-induced range shifts of the two alpine ‘glasshouse’ *Rheum* specie*s*

**Figure 4:**
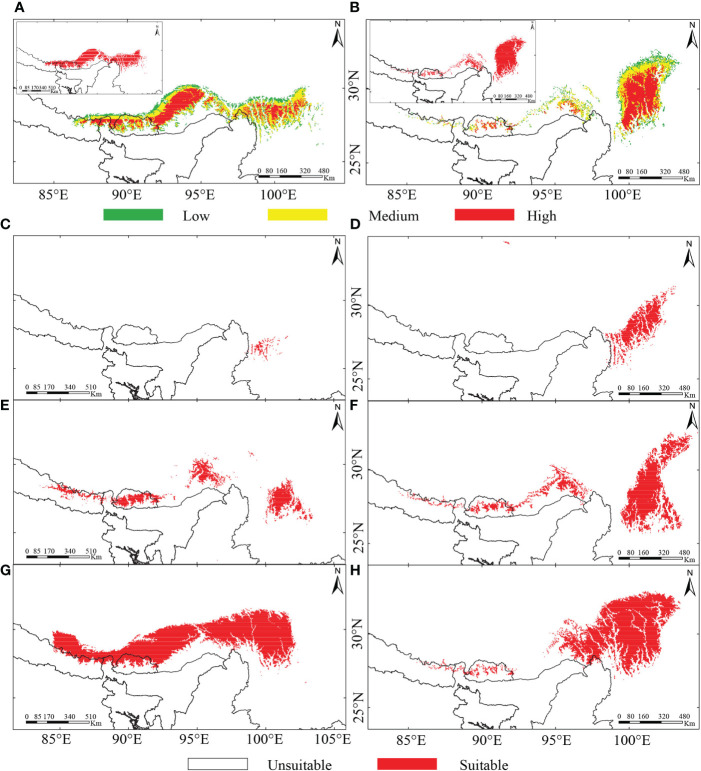
Predicted potentially suitable habitat of two alpine ‘glasshouse’ herbs, **(A, C, E, G)** *Rheum nobile* and **(B, D, F, H)** *Rheum alexandrae* under **(A, B)** present-day scenario of bioclimatic variables classifying their suitability level (suitable habitat in the left top corner of each map), **(C, D)** last-inter glacial (LIG), **(E, F)** last glacial maximum (LGM), and **(G, H)** future scenarios.

The predicted suitable habitat under the initial bioclimatic modeling inferred the better suitability of *R. nobile* across the EH than the HM (**Figure 4A**). On the other hand, *R. alexandrae* has a robust distribution range in the eastern HM and is scattered across the EH (**Figure 4B**). The projected suitability using all environmental variables coupled with bioclimatic variables (second modeling) (**Supplementary Figures S4A, G**) or bioclimatic + each environmental variable (third modeling) (**Supplementary Figures S4B–F, H–L**) resulted in similar patterns for the distribution ranges. The continuous pattern of the distribution range of *R. nobile* runs from the EH to the HM (**Supplementary Figures S4A–F**). In contrast, all three modeling approaches revealed a narrower distribution range of *R. alexandrae* restricted in the HM (**Supplementary Figures S4G–L**). The conservative prediction of high suitability in the HM for both species represents the present-day distribution range of the species (**Figures 1C, D** and **4A, B** **;** **Supplementary Figure S4**). Although they appear in distinct locations, their range comprises the same geographic region in the HM, namely Huluhai and Hongshan (field observation) (**Figures 1B–D**).

**Figure 5:**
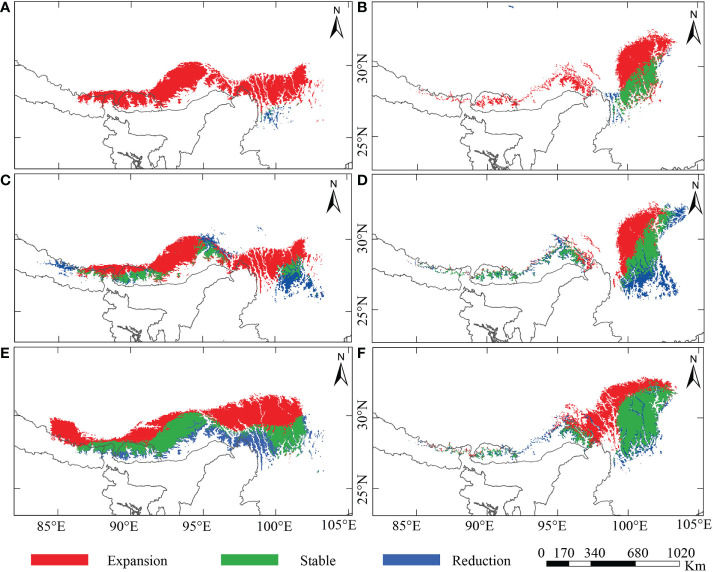
Potentially reduced, stable, and expanded range for the alpine ‘glasshouse’ herbs, **(A, C, E)** *Rheum nobile* and **(B, D, F)** *Rheum alexandrae* compared among different climate scenarios: **(A, B)** last interglacial (LIG) *versus* present-day; **(C, D)** last glacial maximum (LGM) *versus* present-day; and **(E, F)** present-day versus future [2070, Representative Concentration Pathway (RCP) 4.5].

The prediction under four different climate scenarios (LIG, LGM, present-day, and future) are mainly observed across the Eastern Himalayas and the Hengduan Mountains (**Figures 1C, D** and **4** **;** **Supplementary Figure S4**). From LIG to the present-day, *Rheum nobile* has a varied suitable distribution range, whereas a future range is comparable with the present-day (**Figures 4A, C, E, G**). Throughout LIG, the paleo-distribution model predicted refugial habitats in the southern HM (**Figure 4C**), which relocated east and west in distinct regions during the LGM (**Figure 4E**). Similarly, *R. alexandrae*’s suitable distribution expands from the LIG to the present-day and then to the future (**Figures 4B, D, F, H**). *R. alexandrae* was more common in the central HM during the LIG than *R. nobile*. Then, the distribution range shifts towards the eastern HM, with some patches towards the EH during the LGM. When projected to the future scenario, the suitable range in the northern HM moves even higher (**Figure 4H**). Both species were predicted to have suitable distribution ranges and a strong expansion towards the north (**Figure 5**). However, their distribution ranges were only slightly reduced from the paleo climatic scenario [LIG (**Figures 5A, B**) and LGM (**Figures 5C, D**)] to the present-day, and from the present-day to the future (**Figures 5E, F**). However, in comparison to the present-day situation, the expansion towards the north is accompanied by a habitat contraction in the south, notably in the HM. Conclusively, the ensemble forecasting for the two species shows different range shifts under different climatic scenarios.

## Discussion

### Predictiveness of the multi-model median for eSDM

Climate change in mountainous regions is projected to shift plant distribution upwards, consequently, declining their populations in those areas where it is no longer suited for them. The capacity to model such alterations in plant distribution ranges is heavily reliant on the availability of sufficient environmental data and algorithms to analyze the temporal changes. Ensemble modeling has proven to be robust and predictive, overcoming the uncertainty and variability of single models across multiple GCMs (Murphy et al., 2004; Pierce et al., 2009; Rana et al., 2021). The use of ensemble modeling in conjunction with consensus projections not only reduces the predictive uncertainty of single models (Araujo and New, 2007; Ranjitkar et al., 2014) but also improves accuracy (Marmion et al., 2009) and addresses uncertainties inherent in SDM techniques (Barry and Elith, 2006). However, it is equally important to consider the selective nature of variables selected through a series of variable filtration used in the different modeling. Our study is the first to use an ensemble forecasting procedure in an assemblage of variables related to bioclimatic, geo-climatic, habitat heterogeneity, the number of growing days, landcover, and ultraviolet radiation to predict the range shift of plant species. It is anticipated to have a comparable predictive value when forecasting the range shift of montane plants based on only bioclimatic variables (Rana et al., 2021). Nonetheless, alpine plants must use bioclimatic variables with macro/micro-climatic environmental variables, habitat heterogeneity, landcover, canopy cover, and UV radiation (Austin and van Niel, 2011; Robinson et al., 2018). As a result, model uncertainty is minimized, and the algorithm’s accuracy is improved. We analyzed and evaluated the algorithms using threshold-independent (i.e., AUC) and threshold-dependent (i.e., TSS and Cohen’s kappa) model performance measures and outlined the accuracy and predictiveness (matrices values >0.9) of algorithms in different eSDM for both species.

### Ecological niche characterization of the two alpine ‘glasshouse’ *Rheum* species

Global warming will significantly impact the distribution and resilience of the earth’s ecological communities (Mclaughlin and Zavaleta, 2012). These impacts will most likely be felt in mountainous regions, as many alpine plants’ ranges are projected to migrate upwards/polewards (IPCC, 2014). Meanwhile, we found that the distribution of alpine plants, such as the two ‘glasshouse’ *Rheum* species, are characterized by precipitation-dependent variables more than temperature-dependent variables, thereby confirming Fu et al. (2018) and Sun et al. (2016). Along with that, the climatic niche for *R. nobile* is also characterized by a ‘temperature annual range’ with peak suitability at ca. 28˚C, higher than Wang (2006) estimation (i.e., 15 ˚C). *Rheum alexandrae*, on the other hand, has a ‘mean temperature of warmest quarter’ of ca. 8 ˚C. Therefore, the temperature-induced shift in bioclimatic zones is the decisive factor responsible for the decline in the suitability range of the two alpine species. However, their local occurrence and ecological microhabitat are frequently associated with soil water conditions. *Rheum alexandrae* is found chiefly in alpine wetlands, including marsh, swampy meadows, and lakeshores (Chen, 1993); while *R. nobile*, usually occurs on alpine scree and rarely in open patches of alpine meadow, i.e., in well-drained habitats (Song et al., 2013a; Song et al., 2013b).

Specifically, climate regulates the distribution limitations of the two *Rheum* species. The species’ cool range limits are governed directly by climatic factors, while the warm range limits are more impacted by biotic interactions, such as predation, mutualism, and resource competition (Paquette and Hargreaves, 2021). Furthermore, our model exemplifies the hypothesis of a combined latitudinal/upward range shift of species with cool range limitations. Despite their close relationship and similar morphology (Sun et al., 2012; Song et al., 2020b), *R. alexandrae* and *R. nobile* exhibit distinct life histories, distribution limits, and ecological niches related to soil water conditions. Consequently, their specific local niches might underpin the biogeographic patterns of alpine ‘glasshouse’ species. Their specific areas of occurrence were barely apart from each other. They are in comparable geographical places. However, they had never existed together in the same microhabitat. Consequently, niche identity and background analyses for the two ‘glasshouse’ species revealed minimal evidence of comparable niche composition.

### Global warming affects the distribution range of the two alpine ‘glasshouse’ *Rheum* species

Will species be able to adapt in the face of distribution range shift as a result of climate warming? Such topics have been hotly debated, but it is still poorly understood in science. Many researchers argue that species that have previously adapted to worsening climate conditions may be threatened more seriously. In contrast, others fear that the shift in species ranges caused by global warming may result in species extinction (Williams et al., 2007). For mountainous plant species, global warming is expected to act as an ‘escalator to extinction’ (Freeman et al., 2018). This is especially true for species living near mountaintops that cannot move higher. Our model illustrates the range shifts due to global warming for the two alpine ‘glasshouse’ *Rheum* species. The model identified their distinct niches, influencing their responses to predicted climate change and their shift across the Himalayas and the Hengduan Mountains (You et al., 2018). Further, our model suggested that the appropriate range for *Rheum* species has risen since the past LIG, as a result of climate change. The existence of suitable habitats in the HM for both the species during glacial-interglacial periods, especially for *R. alexandrae*, reflects that the HM was not completely glaciated and likely served as a refugium for many plants during glacial periods (Qiu et al., 2011; Zhang et al., 2018; Muellner-Riehl, 2019). The specific nature of the habitat niches for the two *Rheum* species led to an increased suitable range from LIG to LGM.

The southern HM provided a refugium for *R. nobile* and the southeast HM for *R. alexandrae*. As a result of global cooling, the eSDM suggested that both species were able to expand their geographic range from LIG to LGM in different magnitudes (Retallack, 2001; Meng et al., 2017). Possibly, glaciations during the LGM might have pushed the suitable range of *R. nobile* towards the EH and east of the HM. At the same time, *R. alexandrae*, during the LIG, slightly retreated its area of occurrence towards the southern HM. However, an occasional long-distance dispersal of the seed by wind (Song et al., 2013b) may have shaped the current HHM distribution for *R. nobile*. The present-day model of the suitable distribution range represents the actual distribution for both species, except for that of *R. alexandrae* in the EH. *Rheum nobile* is predicted to have a wide suitable range in the EH and the HM, but for *R. alexandrae* only in the HM. The HHM endemic *R. nobile* conjunctly occurs in the HM (especially in the Huluhai and Hongshan regions) but in the distinct areas from *R. alexandrae*.

The present-day similar distribution ranges of the two species in the EH and the HM will no longer exist under climate warming (**Figures 4**, **5**). We speculate that super-competitor species that increase their range northward may benefit from identical niche requirements due to climate change (Liang et al., 2018; Rumpf et al., 2018). Species’ ability to adapt genetically or modify their physiological tolerance will determine their local survival. The dramatic temperature fluctuation during the quaternary glacial cycles has led plant species to move downslope during the LGM and then return upslope with the Holocene warming (Flenley, 1998; Bush et al., 2004). Similarly, since glacial-interglacial periods, our model of warming scenarios predicted north- and upwards expansion of suitable habitats in the HHM and retreat of suitable habitats in the south. The complex topography in the high HM provides enough land surface, allowing upward relocation of suitable habitats and a northward expansion into adjacent areas with suitable habitats (Liang et al., 2018). Similar results were found in SDM studies for other Himalayan species (Song et al., 2004; Xiaodan et al., 2011; Zhao et al., 2011; Manish et al., 2016; Lamsal et al., 2017), which predicted a northward vegetation shift and re-shuffling of plant assemblages in the future due to global warming. Apart from that, comparable findings were observed for Australian plants (Auld et al., 2022), along with other mountain species in the European Alps (Thuiller et al., 2005; Benito et al., 2011; Dullinger et al., 2012), the Andes (Ruiz et al., 2008; Feeley and Silman, 2010), and in arctic regions of the northern hemisphere (Kaplan and New, 2006). However, such range shifts may not be sufficient to ensure population and species survival (Rana et al., 2017). Alpine species may need to change physiologically to cope with the specific climatic circumstances found at the higher elevations (Körner, 2003); seeds, in particular, must be viable for germination and resistant to the ‘summit trap phenomena’ (Pertoldi and Bach, 2007). A failure to adapt might cause mountaintop species to face the risk of ‘mountaintop extinction’ unless they have disjunct populations elsewhere on higher mountains or in colder latitudes (Williams et al., 2007; Lenoir et al., 2008).

### Conservation implication in response to the rise in global temperature

Local extinction of mountain species is a significant driver altering ecosystem structure and misbalancing biodiversity in hotspots. The Himalayas and the Mountains of Southwest China (also known as the HM) are two crucial biodiversity hotspots (Myers et al., 2000; Mittermeier et al., 2011) with a high proportion of endemic mountaintop species. Endemic high mountain species like *R. nobile* and *R. alexandrae* are more vulnerable to local extinction due to global warming as their distribution range is pushed higher and higher. The species’ ability to survive in extreme conditions may be jeopardized if its distribution range expands towards mountaintops. Instead, despite having the adaptive capacity to endure challenging conditions at the mountaintops, the species must suffer local extinction due to increased elevational range contraction (Freeman et al., 2018). Therefore, a centerpiece of biodiversity conservation always warrants the conservation of remaining habitats from further loss.

Moreover, the quantification and mapping of the suitable range of the alpine ‘glasshouse’ *Rheum* in response to climate change have implications for conservation policies aimed at protecting their natural habitats. Our future-predicted map of suitable habitats can be used to identify critical habitat areas and prioritize conservation requirements, reducing biodiversity loss due to habitat degradation and loss. As a result, given the current period of biodiversity loss, there is a desperate need to understand the mechanisms driving the biodiversity dynamics in hotspots, as these findings will aid in the development of conservation policies to safeguard species threatened by environmental changes and human influences. We know that conservation efforts will be challenged by the severity of climate change and the different range shifts of certain species due to climate change (Williams et al., 2007). Furthermore, rising anthropogenic pressure from increased root harvesting for Tibetan medicinal purposes (Song et al., 2020b) has enhanced the need for conservation. Additionally, grazing pressure by yaks has dramatically increased in grassland during summer and autumn (Yang and Du, 1990). It is likely to pose a threat to the rare *R. nobile* even by moderate grazing and thus this flagship species could be endangered by intensified traditional pastoralism (Song et al., 2020b). The information presented here about future climate refugia should be utilized to drive the establishment of conservation areas and conservation initiatives.

## Conclusions

Our research investigates the range shift pattern of two alpine ‘glasshouse’ species, *R. nobile* and *R. alexandrae*, under different climate change scenarios using different eSDM. We explored climatic variables in conjunction with environmental variables to forecast the species’ geographical distribution range. This research demonstrated the importance of applying eSDM and multi-matrices model evaluation methodologies for robust alpine plant range shift projections. Our model predicted the two *Rheum* species’ northward and upward migration from the last glacial period to a future climate change scenario. We not only predicted a shift in the two focus species’ distribution ranges but also exemplified the sympatric coexistence of two *Rheum* species, albeit in distinct ecological niches. Despite having evolved to deal with extreme alpine climatic conditions, the predicted distribution range north- and upwards of the two *Rheum* species may not ensure their survival in the future. Our findings highlight the necessity of combining mechanistic research with knowledge of the physiological and molecular mechanisms that underpin distribution range and current biodiversity patterns. In this era of accelerating biodiversity loss, it is critical to understand the mechanism that drives biodiversity dynamics in hotspots. Through eSDM, we argue for a better understanding of alpine plants’ species range shifts and better biogeographer insight into adaptations and biodiversity conservation objectives.

## Data availability statement

The datasets presented in this study can be found in online repositories. The names of the repository/repositories and accession number(s) can be found below: Dryad depository (https://doi.org/10.5061/dryad.jq2bvq8b3).

## Author contributions

HS, SKR and BS conceived the ideas. SKR, BS and HKR collected the data. SKR and HKR analyzed the data with the support of JS, SR; and SKR wrote the manuscript with the support of HS, BS, JS, HKR, and SR. All authors contributed to the article and approved the submitted version.

## Funding

The first author is supported by the ‘CAS President’s International Fellowship Initiative’ (PIFI) postdoctoral fellowship (2021PB0034). This study was funded by the Second Tibetan Plateau Scientific Expedition and Research (STEP) program (2019QZKK0502), the Strategic Priority Research Program of the Chinese Academy of Sciences (XDA20050203), the National Natural Science Foundation of China (31770249, 32071669 and 32150410356), the Key Projects of the Joint Fund of the National Natural Science Foundation of China (U1802232).

## Acknowledgments

The authors thank numerous collaborators for their contribution to gathering the occurrence of the *Rheum* species, especially curators of the Herbarium of the Kunming Institute of Botany, CAS (KUN) and the National Herbarium and Plant Laboratories, Nepal (KATH).

## Conflict of interest

The authors declare that the research was conducted in the absence of any commercial or financial relationships that could be construed as a potential conflict of interest.

## Publisher’s note

All claims expressed in this article are solely those of the authors and do not necessarily represent those of their affiliated organizations, or those of the publisher, the editors and the reviewers. Any product that may be evaluated in this article, or claim that may be made by its manufacturer, is not guaranteed or endorsed by the publisher.

## Supplementary material

Supplementary methods _ODMAP Protocol.

Supplementary Figure 1 Response curves of the highly predictive top two algorithms showing the probability of presence and suitability (y-axis) under the **(A–C, I–K)** temperature-dependent and **(D–H, L–O)** precipitation-dependent variables (x-axis) for **(A–H)** *Rheum nobile* and **(I–O)** *Rheum alexandrae* under the function of ENMTools. The predictive algorithms are the Generalised Additive Model (GAM; red line) and Random Forest (RF; green line). Temperatures are expressed in °C (degree Celsius) and precipitation in mm (millimeter). Refer to  for the bioclimatic variables.

Supplementary Figure 2
**(A)** The scree plot representing the variance explained by the first ten principal components of predictive environmental variables. **(B, C)** The contribution of top predictive environmental variables under the PC1 **(B)** and PC2 **(C)** scores. The Red dashed line indicates the expected average contribution if variable contributions were uniform. Refer to for abbreviated environmental variables.

Supplementary Figure 3 Pairwise niche **(A, B)** identity test and background test **(C, D**, symmetric; **E**, **F**, asymmetric test**)** for the highly predictive top two algorithms **(A, C, E)** Generalized additive model (GAM) and **(B, D, F)** Random forest (RF) model, between the two alpine ‘glasshouse’ herbs *Rheum nobile* and *Rheum alexandrae*, calculated as a function of *niche.overlap* ecospat test using ENMtools. The niche equivalency test as species-wise pair comparisons was measured by Schoener’s *D* (in black color) and Hellinger’s-based *I* (in green color) indices.

Supplementary Figure 4 Predicted potentially suitable habitat of two alpine ‘glasshouse’ herbs, **(A–F)** *Rheum nobile* and **(G–L)** *Rheum alexandrae*, under the present-day scenario of predictive **(A, G)** environmental variables, **(B, H)** geo-climatic, **(C, I)** habitat heterogeneity, **(D, J)** growing days, **(E, K)** ultra-violet radiations, and **(F, L)** consensus landcover.
