# Supplementary material for: Global warming pushes the distribution range of the two alpine ‘glasshouse’ Rheum species north- and upwards in the Eastern Himalayas and the Hengduan Mountains
Source: Front Plant Sci. 2022 Oct 7;13:925296. doi: 10.3389/fpls.2022.925296 (PMC9585287; doi:10.3389/fpls.2022.925296)
Supplement: Supplementary methods — _ODMAP Protocol. [file Table_11.docx]

Global warming pushes the distribution range of the two alpine ‘glasshouse’ Rheum species north- and upwards in the Eastern Himalayas (EH) and the Hengduan Mountains (HM)

– ODMAP Protocol –

xxxxxxxxxxxxxxxxxxxxxxxxxxxxxxxxxx

2022-01-08

## Overview

#### Authorship

Contact : [xxxxxxxxxxxxxxxxxxx](mailto:santosh@mail.kib.ac.cn)

Study link: In review

#### Model objective

Model objective: Forecast and transfer

Target output: continuous habitat suitability, distribution range shift

#### Focal Taxon

Focal Taxon: *Rheum nobile* and *Rheum alexandrae*

#### Location

Location: The Eastern Himalaya (EH) and the Hengduan Mountains (HM)

#### Scale of Analysis

Spatial extent: 50, 160, 0, 60 (xmin, xmax, ymin, ymax)

Spatial resolution: ~5 km (2.5 arc minutes)

Temporal extent: LIG (last inter-glacial c. 120,000–140,000 years BP), LGM (last glacial maximum c. 22,000 years BP) and future (2070)

Boundary: natural

#### Biodiversity data

Observation type: GPS tracking, field survey

Response data type: point occurrence, presence/absence

#### Predictors

Predictor types: climatic, habitat

#### Hypotheses

Hypotheses: We hypothesized that the global warming pushes the distribution range of alpine 'glasshouse' Rheum species.

#### Assumptions

Model assumptions: We assumed that the gathered presence points and multi-steps multi-phase multi-median model eSDM provides the robust prediction of the suitable habitat range.

#### Algorithms

Modelling techniques: maxent, glm, mars, ann, gam, fda, randomForest

Model complexity: Multi-phase ensemble species distribution modelling (eSDM) have been found to outperform single models and single scaled models with robust prediction. Therefore, we preferred to incorporate multi-steps variables selection under multi-median model of GCMs for multi-phase eSDM into our analyses.

Model averaging: We created an ensemble model by taking the weighted average of each model’s predicted surface proportional to the TSS/Kappa/AUC value of model performance.

#### Workflow

Model workflow: Model workflow is provided below. Full methodological details can be found in the Methods section of the paper. 1) The spatially rarefied occurrence points of the focal species Rheum nobile and Rheum alexandrae were compiled from field surveys, herbarium records, and available online database. The random background points were selected within an area of 25–50 km radius from the point of occurrence following Barbet-Massin et al. (2012). 2) We adopted the novel three phase eSDM through three steps predictive variables selection under different environmental strata. The environmental strata are geo-climate related, habitat heterogeneity, growing days, ultra-violet radiation, and consensus land-cover. Besides the current and LIG bioclimatic variables, we used multi-median model of different GCMs for paleoclimatic (LGM) and future climatic scenarios with a spatial resolution of 2.5 arc-min. 3) We identified predictive variables through multi-steps variable selection through pairwise Spearman’s rank correlations and Variance Inflation Factor (VIF). The final subset of explanatory variables includes criterion with the Pearson correlation values < |0.8| and VIF*j* values < 10. 4) An ensemble of species distribution modelling was implemented using ‘Biomod2’ R-package. The assessment of eSDM on niche-based modelling techniques allows performing different modelling approaches as bioclimatic envelopes, regression, classification methods, and machine learning methods. The consistent modelling approach was used throughout the multi-phase strategies implemented for R. nobile and R. alexandrae. Moreover, the niche equivalency test was analysed using ecospat test in ‘ENMTools’ R-package among the two focal species, R. nobile and R. alexandrae. 5) We used this model to predict the range shift pattern of the suitable habitat of two focal species. 6) The current prediction of the model was then forecasted into paleoclimatic (LIG, LGM) and future (2070, RCP4.5) climatic scenarios. The spatial conversion of the consensus ensemble model to a binary model (presence/absence) was based on the thresholds (50% of the suitable habitat) that suits the current distribution of the focal species. All the spatial analyses were implemented in ArcMap 10.4.1 under the extension Spatial Analysis to reclassify changes in LIG, LGM and future conditions compared to current suitability into reduction, stable and expanded areas of the focal species.

#### Software

Software: BIOMOD2 (v.3.4.12) package in the R-programming language (R v.3.6.3)

Code availability: No code links available

Data availability: No code links available

## Data

#### Biodiversity data

Taxon names: *Rheum nobile*, *Rheum alexandrae*

Taxonomic reference system: species: *Rheum nobile* Hook. f. & Thomson; *Rheum alexandrae* Batalin

Ecological level: communities, populations

Data sources: Refer to text for detail data source.

Sampling design: The overfitting or biasness in the eSDM occur mainly due to the geo-coding errors from herbarium label and spatially clustered localities. Therefore, we spatially rarefied these occurrence points to reduce model bias. The occurrence points were spatially rarefied along 10-km spatial grid due to its filter distances’ utility in spatially high heterogeneous regions, like mountains. Out of 79 and 77 records collected for *R. nobile* and *R. alexandrae*, only 56 and 54 occurrence points were retained for building the models, respectively. Then after, we extracted the information’s from the different strata of environmental variables at each of those occurrence points. The strata of environmental variables are bioclimatic variables, geo-climate related variables, habitat heterogeneity, growing days, ultra-violet radiations, and consensus landcover. The bioclimatic variables are used for four different climatic scenarios of LIG (last interglacial), LGM (last glacial maximum), current, future (2070).

Sample size: Out of 79 and 77 records collected for *R. nobile* and *R. alexandrae*, only 56 and 54 occurrence points were used for building the models, respectively.

Clipping: North-east Asia

Cleaning: We spatially rarefied the occurrence points to reduce model bias along 10-km spatial grid due to its filter distances’ utility in spatially high heterogeneous regions, like mountains.

Absence data: Pseudo-absence points were randomly sampled across the study area with a rule that no sampled point could be within 10km from another sampled point. This resulted in 1000 pseudo-absence points for analysis.

Background data: see above

#### Data partitioning

Training data: We defined 4-fold cross-validation using 75% of the data for training the models in Biomod2.

Validation data: We applied the four-step modelling process in ‘Biomod2′ package for the consensus mapping. Firstly, we calibrated the 10 sub-models applying ‘BIOMOD_Modelling’ function defining 4-fold cross-validation using 75% of the data for training the models. The remaining 25% were used for evaluating the predictive power using True Skill Statistics (TSS), Cohen’s Kappa and Area Under Curve-Receiver Operating characteristics (AUC) statistics. Secondly, we applied the ‘BIOMOD_EnsembleModeling’ function with sub-model that weights > 0.6 evaluated by TSS, Cohen’s kappa, AUC retained for ensemble modelling. The sampling procedure was replicated 5 times. Thirdly, we used ‘BIOMOD_Projection’ for projecting the calibrated sub-models within Biomod2 into new space or time. The last step was ‘BIOMOD_EnsembleForecasting’ function to generate the consensus mapping of species over space and time.

Test data: The remaining 25% were used for evaluating the predictive power of the test data.

#### Predictor variables

Predictor variables: Bioclimatic variables (Mean diurnal range, Isothermality, Temperature Annual Range (bio5-bio6), Mean Temperature of Wettest Quarter, Mean Temperature of Warmest Quarter, Precipitation of Wettest Month, Precipitation Seasonality, Precipitation of Wettest Quarter, Precipitation of Driest Quarter, Precipitation of Coldest Quarter); Geo-climate related (Elevation, Aspect, Net primary productivity, Annual aridity, Soil moisture, Potential evapotranspiration, Annual Relative Humidity, Annual solar radiation, Soil pH, Soil carbon, Annual water vapour); Habitat heterogeneity (Correlation, Coefficient of variation, Evenness, Homogeneity, Maximum, Range, Contrast); Growing days (Growing degree days, Growing season length, Growing season temperature, Last day of growing season); Ultra-violet radiations (UV-B seasonality, Mean UV-B of lowest month, Sum of monthly mean UV-B during lowest quarter); Consensus landcover (Evergreen/deciduous Needleleaf trees, Mixed/other trees, Shrubs, Herbaceous vegetation, Cultivated and managed vegetation, Snow/ice).

Data sources: Refer to text for detail data source.

Spatial extent: 50, 160, 0, 60 (xmin, xmax, ymin, ymax)

Spatial resolution: 2.5 arc min.

Coordinate reference system: CGS_WGS_1984

Temporal extent: 1990 - 2021

## Model

#### Variable pre-selection

Variable pre-selection: The pre-selection of variables were based on the biological significance of the micro-habitat of both the focal species through field observations.

#### Multicollinearity

Multicollinearity: We applied series of variable selection for the multi-steps ensemble forecasting. Firstly, variable were selected categories-wise ; secondly, all categories combinedly (for combined ensemble forecasting); and thirdly, bioclimatic variables with other environmental variables categories. Initially screened global consensus landcover variables was analyzed with occurrence points greater than 30% threshold out of total occurrence points for predictive variable selection of the focal species. As the main core of variable selection, we retained variables with variance inflation factor (VIF<10) and Pearson correlation (r<|0.8|).

#### Model settings

maxent: note (All default settings in Biomod2 were used except maximum iterations = 5000.)

glm: note (All default settings in Biomod2 were used.)

mars: note (All default settings in Biomod2 were used.)

ann: note (All default settings in Biomod2 were used.)

gam: note (All default settings in Biomod2 were used.)

fda: note (All default settings in Biomod2 were used.)

randomForest: note (All default settings in Biomod2 were used.)

#### Model selection - model averaging - ensembles

Model selection: The algorithms/model were selected based on TSS, Cohen's kappa and AUC

Model averaging: We used sub-model that weights > 0.9 evaluated by TSS

Model ensembles: The ensemble model were finally selected based o TSS, Cohen's kappa and AUC >0.9

#### Analysis and Correction of non-independence

Spatial autocorrelation: We addressed the spatial autocorrelation of the occurrence points by filtering the points using 10 km spatial grid, where each grid possess only one points.

#### Threshold selection

Threshold selection: The consensus ensemble model was spatially converted to a binary model (presence/absence) applying thresholds that allow a maximum of 50% habitat suitability that suits the current distribution of the focal species.

## Assessment

#### Performance statistics

Performance on training data: TSS

Performance on test data: TSS, Kappa, AUC

#### Plausibility check

Response shapes: The response plots of highly predictive top three algorithms/sub-model were plotted showing the probability of presence under the highly contributed bioclimatic variables implemented in Biomod2 package. Besides, ENMTools was used to plot the response of bioclimatic variables for the highly predictive top two algorithms Generalized additive model (GAM) and Random forest (RF) model for the niche test of focal species

Expert judgement: The map was displayed as the function of suitable and unsuitable based on threshold setup for eSDM.

## Prediction

#### Prediction output

Prediction unit: Raster

Post-processing: All the spatial analyses were implemented in ArcMap 10.4.1 under the extension Spatial Analysis to reclassify changes in LIG, LGM and future conditions compared to current suitability into reduction, stable and expanded areas of the focal species. The forecasted suitable habitat maps of two focal species were then overlaid to identify overlapping region under current climatic scenarios, along with the level of suitability (low, medium, and high).

#### Uncertainty quantification

Scenario uncertainty: The uncertainty in scenarios was reduced by adopting the multi-model median (MMM) ensemble of General Circulation Models (GCMs) (Rana et al., 2021). The MMM ensemble of GCMs was obtained for the 19 climatic variables considering the median of 3 GCMs for LGM provided by Paleoclimate Modelling Inter-comparisons Project Phase 5 (Braconnot et al., 2007) and 19 GCMs for future provided by CMIP5. Within each GCMs of the future, there are four RCPs , ranging from RCP 2.6 (aggressive mitigation/lowest emissions) to RCP 8.5 (highest emissions scenario). Out of these four RCPs, the best predictive scenario RCP 4.5 that represents ‘stabilization without overshoot’ beyond 2100 was selected as the future scenario for MMM ensemble of GCMs .
