# Supplementary figures and images for: Global warming pushes the distribution range of the two alpine ‘glasshouse’ Rheum species north- and upwards in the Eastern Himalayas and the Hengduan Mountains
Source: Front Plant Sci. 2022 Oct 7;13:925296. doi: 10.3389/fpls.2022.925296 (PMC9585287; doi:10.3389/fpls.2022.925296)

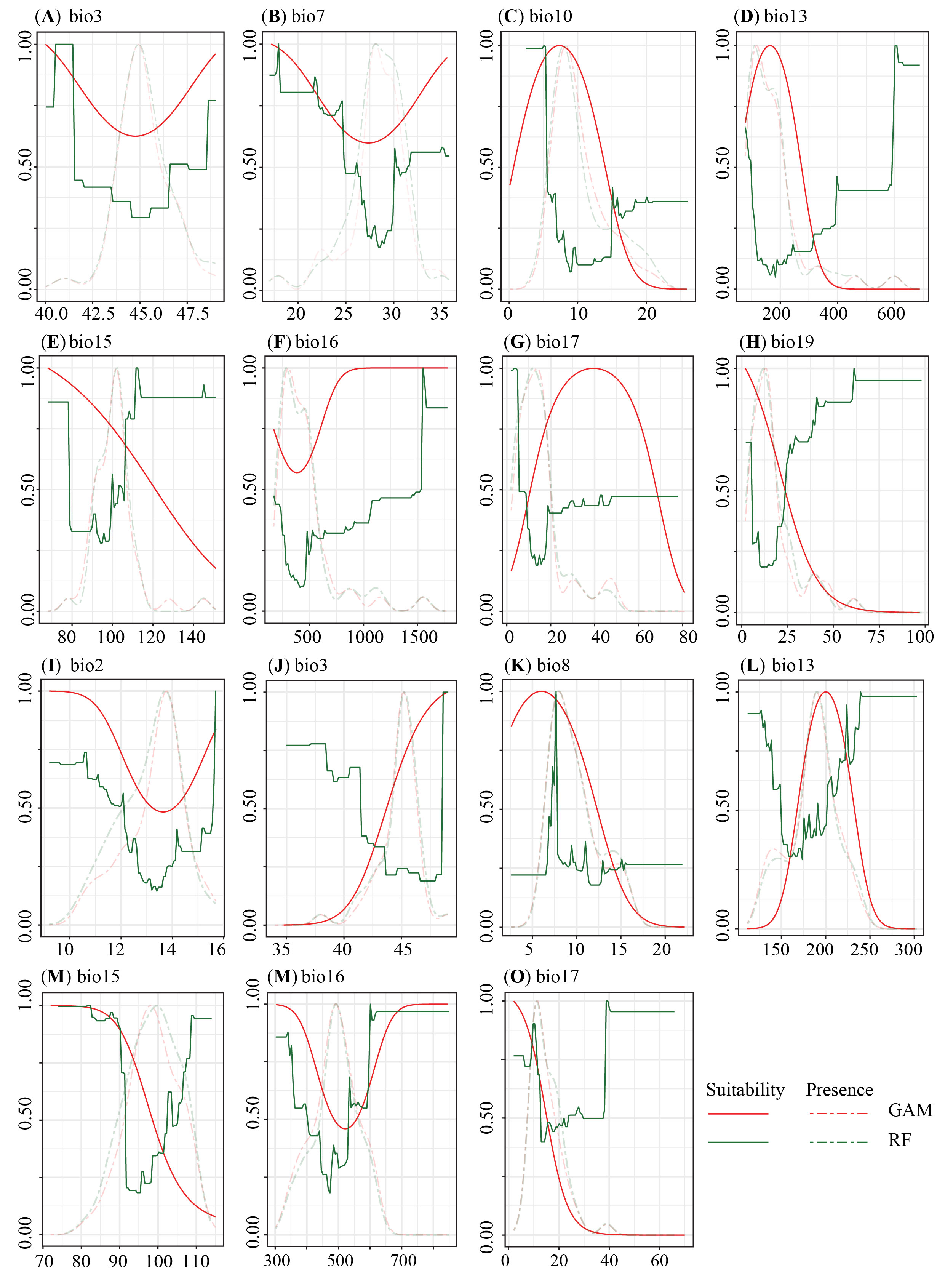

Supplement: Supplementary Figure 1 — Response curves of the highly predictive top two algorithms showing the probability of presence and suitability (y-axis) under the (A–C, I–K) temperature-dependent and (D–H, L–O) precipitation-dependent variables (x-axis) for (A–H) Rheum nobile and (I–O) Rheum alexandrae under the function of ENMTools. The predictive algorithms are the Generalised Additive Model (GAM; red line) and Random Forest (RF; green line). Temperatures are expressed in °C (degree Celsius) and precipitation in mm (millimeter). Refer to for the bioclimatic variables. [file Image_1.jpeg]

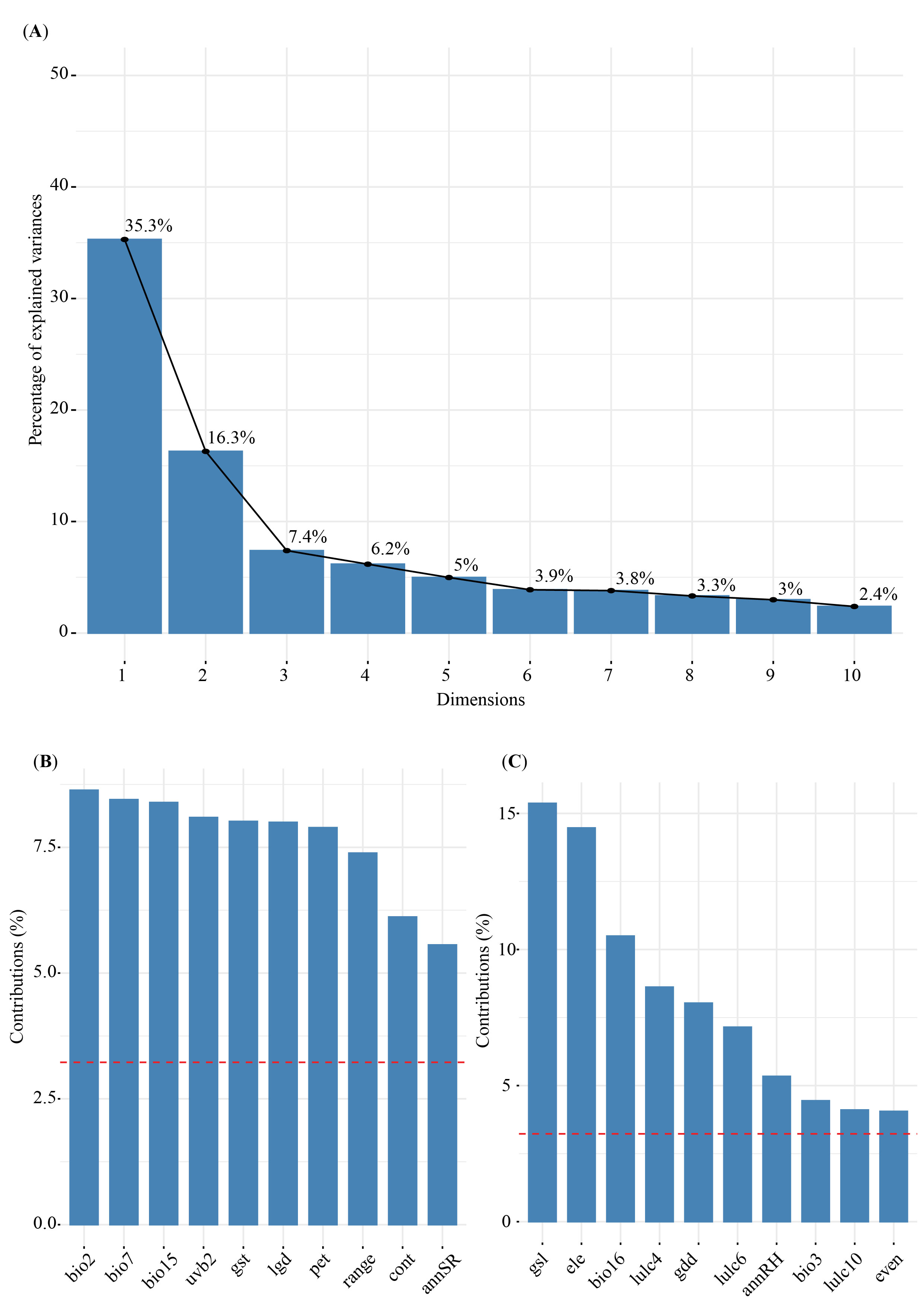

Supplement: Supplementary Figure 2 — (A) The scree plot representing the variance explained by the first ten principal components of predictive environmental variables. (B, C) The contribution of top predictive environmental variables under the PC1 (B) and PC2 (C) scores. The Red dashed line indicates the expected average contribution if variable contributions were uniform. Refer to for abbreviated environmental variables. [file Image_2.jpeg]

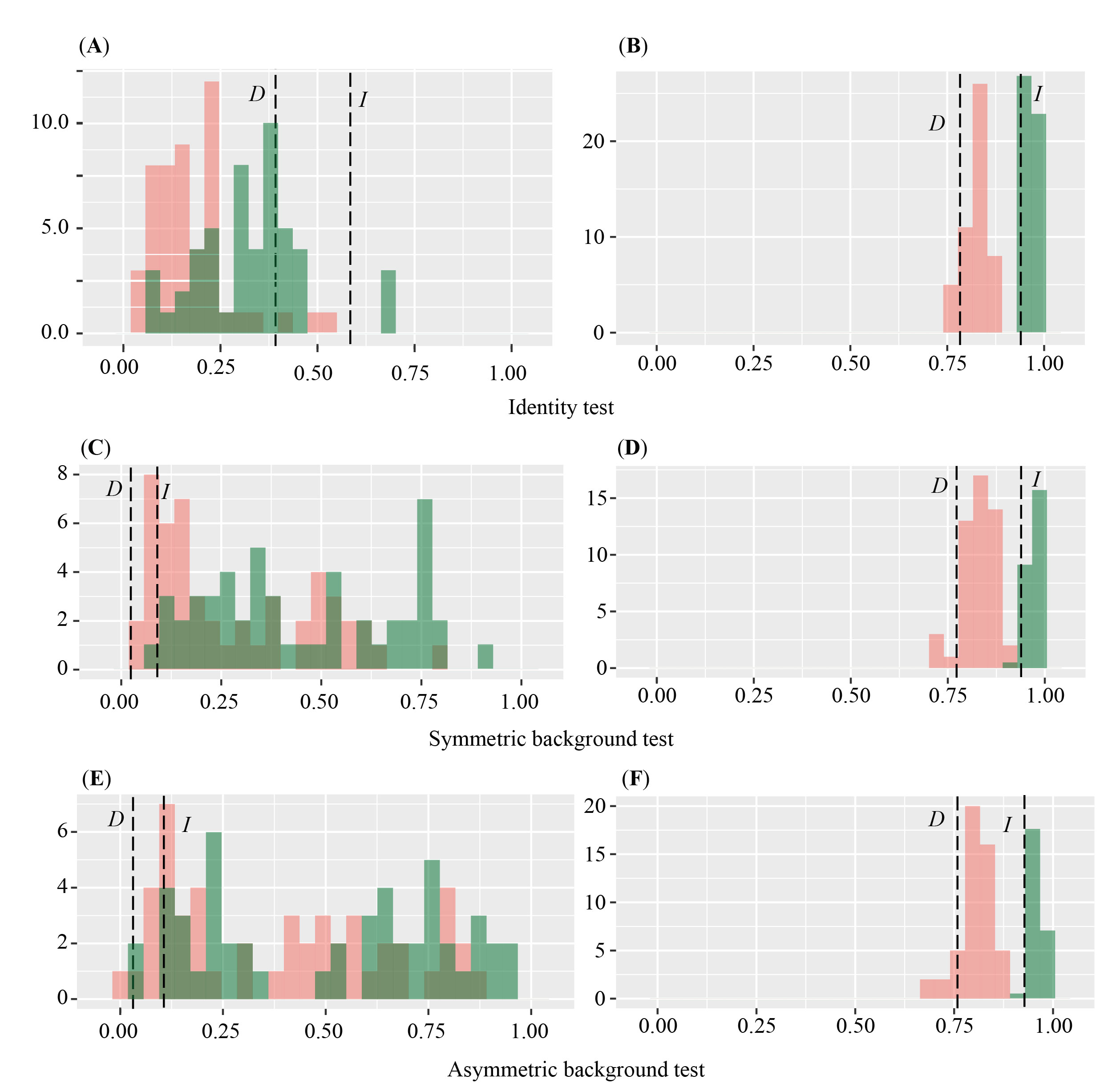

Supplement: Supplementary Figure 3 — Pairwise niche (A, B) identity test and background test (C, D, symmetric; E, F, asymmetric test) for the highly predictive top two algorithms (A, C, E) Generalized additive model (GAM) and (B, D, F) Random forest (RF) model, between the two alpine ‘glasshouse’ herbs Rheum nobile and Rheum alexandrae, calculated as a function of niche.overlap ecospat test using ENMtools. The niche equivalency test as species-wise pair comparisons was measured by Schoener’s D (in black color) and Hellinger’s-based I (in green color) indices. [file Image_3.jpeg]

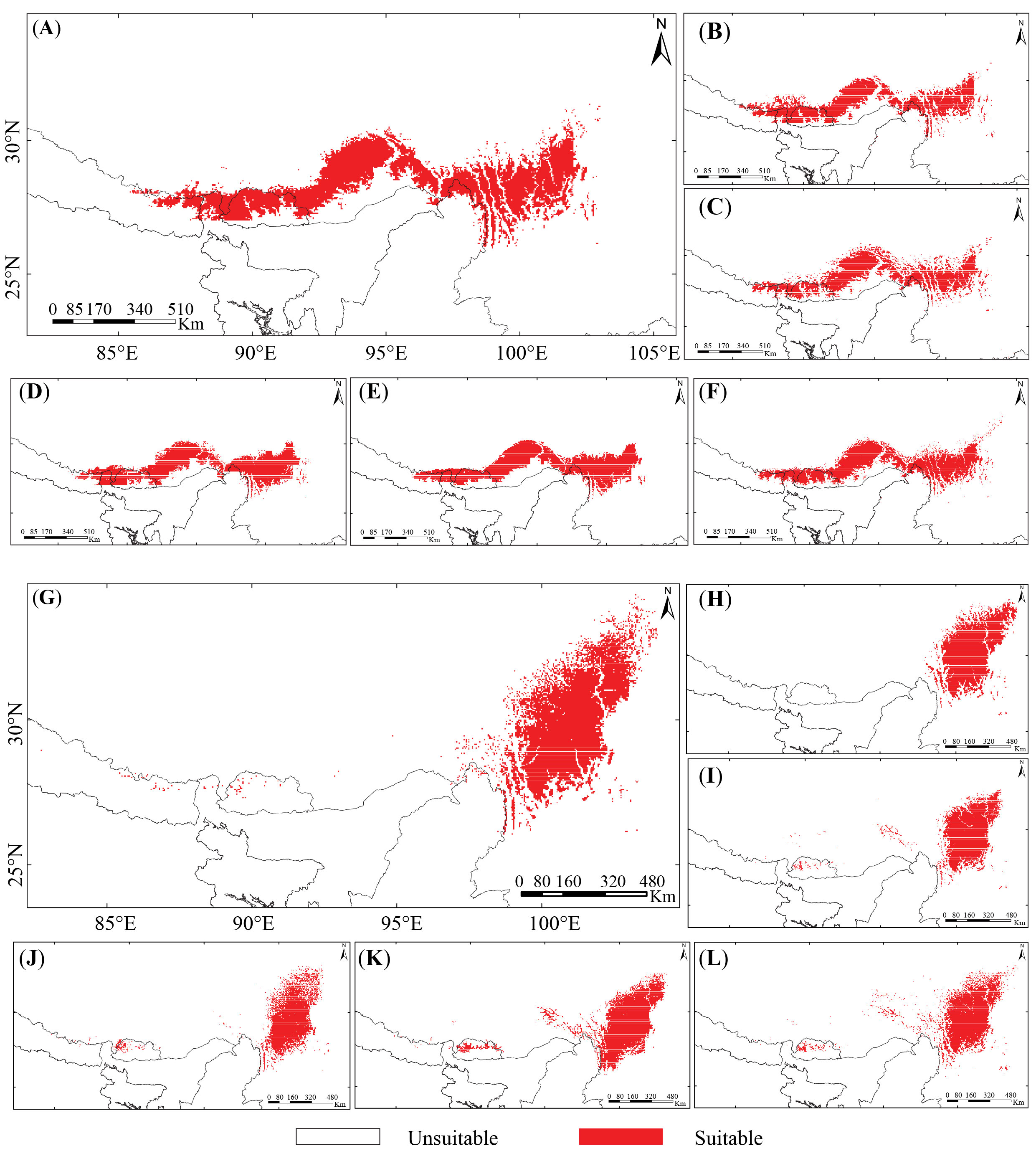

Supplement: Supplementary Figure 4 — Predicted potentially suitable habitat of two alpine ‘glasshouse’ herbs, (A–F) Rheum nobile and (G–L) Rheum alexandrae, under the present-day scenario of predictive (A, G) environmental variables, (B, H) geo-climatic, (C, I) habitat heterogeneity, (D, J) growing days, (E, K) ultra-violet radiations, and (F, L) consensus landcover. [file Image_4.jpeg]
