# Supplementary material for: Global warming pushes the distribution range of the two alpine ‘glasshouse’ Rheum species north- and upwards in the Eastern Himalayas and the Hengduan Mountains
Source: Front Plant Sci. 2022 Oct 7;13:925296. doi: 10.3389/fpls.2022.925296 (PMC9585287; doi:10.3389/fpls.2022.925296)
Supplement: Supplementary file 6 [file Table_1.docx]

**Supplementary Table S1** | List of past field surveys occurrence datasets gathered since 1937–2020 used in ensemble species distribution modelling.

| **species** | **longitude** | **latitude** | **location** | **elevation** | **year** | **reference** |
| --- | --- | --- | --- | --- | --- | --- |
| *Rheum nobile* | 91.66654 | 27.71141 | Zemithang, AP | 3584 | 2017 | Chakraborty et al., 2017 |
| *Rheum nobile* | 92.15621 | 27.5712 | West Kameng, AP | 4146 | 2015 | Saha & Sundriyal, 2015; Tsering and Tag, 2015 |
| *Rheum nobile* | 91.92658 | 27.65196 | Sela Pass, Tawang, AP | 3643 | 2015 | Tsering & Tag, 2015; Gajurel et al., 2015 |
| *Rheum nobile* | 94.08333 | 28.7 | West Siang, Domje La ca. 13 km N Mechuka, AP | 3800-3970 | 2018 | Bharali et al., 2018 |
| *Rheum nobile* | 99.8642 | 28.06667 | Butan |  |  |  |
| *Rheum nobile* | 94.97977 | 29.54564 | Milin, Xizang, China | 4500 | 1983 |  |
| *Rheum nobile* | 95.55801 | 29.32067 | Motuo, Xizang, China | 4400 | 1982 |  |
| *Rheum nobile* | 94.8945 | 29.45105 | Milin, Xizang, China | 4100 | 1982 |  |
| *Rheum nobile* | 99.92517 | 28.72892 | Xiangcheng, Sichuan, China | 4613 | 1981 |  |
| *Rheum nobile* | 89.01617 | 27.78121 | Yadong, Xizang, China | 4900 | 1975 |  |
| *Rheum nobile* | 93.42384 | 29.07123 | Linzi, Xizang, China | 4400 | 1972 |  |
| *Rheum nobile* | 99.95944 | 28.61944 | Shangri-la, Yunnan, China | 4560 | 2002 |  |
| *Rheum nobile* | 99.9426 | 28.19277 | Shangri-la, Yunnan, China | 4665 | 2011 |  |
| *Rheum nobile* | 99.86472 | 28.591 | Shangri-la, Yunnan, China | 4557 | 2011 |  |
| *Rheum nobile* | 93.37489 | 28.95463 | Langxian, Xizang, China |  | 1977 |  |
| *Rheum nobile* | 94.64917 | 29.61806 | Linzi, Xizang, China | 4705 | 2008 |  |
| *Rheum nobile* | 88.88983 | 27.38125 | Yadong, XiZang, China | 4109 | 2009 |  |
| *Rheum nobile* | 94.64175 | 29.62231 | Linzi, Xizang, China | 4661 | 2009 |  |
| *Rheum nobile* | 99.01096 | 26.54829 | Nujiang, Yunnan, China |  | 1978 |  |
| *Rheum nobile* | 99.9075 | 28.59889 | Shangri-la, Yunnan, China | 4660 | 2011 |  |
| *Rheum nobile* | 94.65194 | 29.61444 | Linzi, Xizang, China | 4620 | 2009 |  |
| *Rheum nobile* | 94.64939 | 29.60556 | Linzi, Xizang, China | 4605 | 2009 |  |
| *Rheum nobile* | 98.74449 | 26.42097 | Nujiang, Yunnan, China | 3938 | 2019 |  |
| *Rheum nobile* | 99.92727 | 28.41954 | Shangri-la, Yunnan, China | 4704 | 2016 |  |
| *Rheum nobile* | 99.9645 | 28.52416 | Shangri-la, Yunnan, China | 4700 | 2013 |  |
| *Rheum nobile* | 99.84443 | 28.58527 | Shangri-la, Yunnan, China | 4569 | 2015 |  |
| *Rheum nobile* | 99.86744 | 28.5876 | Shangri-la, Yunnan, China | 4782 | 2012 |  |
| *Rheum nobile* | 99.88456 | 28.14039 | Shangri-la, Yunnan, China | 4489 | 2019 |  |
| *Rheum nobile* | 99.94255 | 28.18293 | Shangri-la, Yunnan, China | 4635 | 2019 |  |
| *Rheum nobile* | 94.93497 | 29.49052 | Milin, Xizang, China | 3964 | 2019 |  |
| *Rheum nobile* | 94.64915 | 29.61866 | Linzi, Xizang, China | 4728 | 2019 |  |
| *Rheum nobile* | 94.68956 | 29.6225 | Linzi, Xizang, China | 4582 | 2019 |  |
| *Rheum nobile* | 92.9293 | 28.66972 | Xizang, China | 4915 | 2018 |  |
| *Rheum nobile* | 93.08414 | 28.63111 | Shanlan, Xizang, China | 4336 | 2017 |  |
| *Rheum nobile* | 87.73208 | 28.49694 | Dingjie, Xizang, China | 4900 | 1990 |  |
| *Rheum nobile* | 89.18711 | 27.71806 | Yadong, XiZang, China | 4900 | 1975 |  |
| *Rheum nobile* | 101.3251 | 28.68278 | Jiulong, Sichuan, China | 4100 | 1937 |  |
| *Rheum nobile* | 94.88306 | 29.63917 | Linzi, Xizang, China | 4663 | 2006 |  |
| *Rheum nobile* | 87.78 | 27.08333 | Banduke, Jaljale, Nepal | 4250 | 1991 |  |
| *Rheum nobile* | 87.63583 | 27.67528 | Banduke, Jaljale, Nepal | 4917 | 2018 |  |
| *Rheum nobile* | 88.0625 | 27.44 | Timbu Pokhari, Panchthar,Nepal | 4561 | 2018 |  |
| *Rheum nobile* | 87.86361 | 27.68917 | Tin Pokhari, Gyabla,Nepal | 4723 | 2018 |  |
| *Rheum alexandrae* | 99.97383 | 28.20816 | Shangri-la, Yunnan,China | 3926 | 2011 |  |
| *Rheum alexandrae* | 100.2439 | 29.49722 | Litang, Sichuan, China | 4389 | 2010 |  |
| *Rheum alexandrae* | 99.985 | 30.17833 | Litang, Sichuan, China | 4162 | 2010 |  |
| *Rheum alexandrae* | 99.33764 | 30.22408 | Litang, Sichuan, China | 3887 | 2011 |  |
| *Rheum alexandrae* | 100.4731 | 29.80444 | Litang, Sichuan, China | 4600 | 2011 |  |
| *Rheum alexandrae* | 99.7155 | 27.51815 | Shangri-la, Yunnan,China | 3920 | 2009 |  |
| *Rheum alexandrae* | 101.8214 | 30.05306 | Kangding, Sichuan,China | 3860 | 2008 |  |
| *Rheum alexandrae* | 100.1125 | 29.34606 | Daocheng, Sichuan,China | 4374 | 1981 |  |
| *Rheum alexandrae* | 100.2815 | 28.68892 | Daocheng, Sichuan,China | 4300 | 1973 |  |
| *Rheum alexandrae* | 100.1769 | 29.41389 | Litang, Sichuan, China | 4598 | 2011 |  |
| *Rheum alexandrae* | 100.2719 | 29.52722 | Litang, Sichuan, China | 4570 | 2011 |  |
| *Rheum alexandrae* | 99.91083 | 31.38639 | Baiyu, Sichuan,China | 4128 | 2011 |  |
| *Rheum alexandrae* | 100.0896 | 29.14913 | Daocheng, Sichuan,China | 4324 | 2011 |  |
| *Rheum alexandrae* | 100.1791 | 29.41586 | Daocheng, Sichuan,China | 4605 | 2011 |  |
| *Rheum alexandrae* | 98.99593 | 26.91513 | Nujiang,Yunnan,China | 3410 | 1964 |  |
| *Rheum alexandrae* | 101.761 | 30.22239 | Kangding, Sichuan,China | 4037 | 2011 |  |
| *Rheum alexandrae* | 99.91467 | 28.15635 | Shangri-la, Yunnan,China | 4400 | 2008 |  |
| *Rheum alexandrae* | 100.1878 | 29.45492 | Daocheng, Sichuan,China | 4039 | 2011 |  |
| *Rheum alexandrae* | 101.9969 | 29.90806 | Luding,Sichuan,China | 3826 | 2013 |  |
| *Rheum alexandrae* | 100.3256 | 29.60406 | Litang, Sichuan, China | 4144 | 2013 |  |
| *Rheum alexandrae* | 99.97631 | 28.24806 | Shangri-la, Yunnan,China | 3858 | 2010 |  |
| *Rheum alexandrae* | 100.2642 | 29.50169 | Litang, Sichuan, China | 4450 | 1982 |  |
| *Rheum alexandrae* | 99.77686 | 27.43105 | Shangri-la, Yunnan,China | 3850 | 1981 |  |
| *Rheum alexandrae* | 101.4874 | 29.31661 | Jiulong, Sichuan,China | 4180 | 1980 |  |
| *Rheum alexandrae* | 100.0804 | 29.28506 | Daocheng, Sichuan,China | 4100 | 1980 |  |
| *Rheum alexandrae* | 100.7845 | 28.79494 | Muli, Sichuan,China | 4620 | 1978 |  |
| *Rheum alexandrae* | 100.7492 | 28.73461 | Muli, Sichuan,China | 4489 | 1978 |  |
| *Rheum alexandrae* | 101.5393 | 29.27634 | Jiulong, Sichuan,China | 4000 | 1974 |  |
| *Rheum alexandrae* | 101.568 | 30.59882 | Daofu, Sichuan,China | 4200 | 1974 |  |
| *Rheum alexandrae* | 100.0036 | 27.8262 | Shangri-la, Yunnan,China | 3500 | 1970 |  |
| *Rheum alexandrae* | 101.2007 | 31.00508 | Daofu, Sichuan,China | 3800 | 1961 |  |
| *Rheum alexandrae* | 101.6951 | 30.85314 | Ganzi, Sichuan,China | 4300 | 1960 |  |
| *Rheum alexandrae* | 101.7747 | 30.0902 | Kangding, Sichuan,China | 4100 | 1953 |  |
| *Rheum alexandrae* | 100.2165 | 27.10375 | Lijiang,Yunnan,China | 3873 | 1957 |  |
| *Rheum alexandrae* | 100.8439 | 29.99833 | Yajiang, Sichuan,China | 4273 | 2011 |  |
| *Rheum alexandrae* | 100.2694 | 29.5366 | Daocheng, Sichuan,China | 4376 | 2009 |  |
| *Rheum alexandrae* | 100.2856 | 28.89917 | Daocheng, Sichuan,China | 4460-4650 | 1998 |  |
| *Rheum alexandrae* | 99.89374 | 28.12849 | Shangri-la, Yunnan,China | 4272 | 2019 |  |
| *Rheum alexandrae* | 101.4567 | 29.23639 | Jiulong, Sichuan,China | 3760 | 2005 |  |
| *Rheum alexandrae* | 101.4069 | 29.15306 | Jiulong, Sichuan,China | 3700 | 2005 |  |
| *Rheum alexandrae* | 101.3081 | 30.04444 | Yajiang, Sichuan,China | 3850 | 2006 |  |
| *Rheum alexandrae* | 101.3875 | 30.025 | Kangding, Sichuan,China | 4250 | 2006 |  |
| *Rheum alexandrae* | 99.5575 | 30.30361 | Batang, Sichuan,China | 4500 | 2006 |  |
| *Rheum alexandrae* | 100.2719 | 29.51694 | Litang, Sichuan, China | 4640-4830 | 2006 |  |
| *Rheum alexandrae* | 100.2583 | 30.21806 | Litang, Sichuan, China | 4400 | 2006 |  |
| *Rheum alexandrae* | 99.86139 | 31.24222 | Baiyu, Sichuan,China | 4510 | 2006 |  |
| *Rheum alexandrae* | 101.6589 | 30.47028 | Daofu, Sichuan,China | 3680 | 2007 |  |
| *Rheum alexandrae* | 99.78013 | 26.64758 | Lijiang,Yunnan,China | 3619 | 2012 |  |
| *Rheum alexandrae* | 99.94328 | 27.90431 | Shangri-la, Yunnan,China | 3620 | 2017 |  |
| *Rheum alexandrae* | 99.91134 | 28.42269 | Shangri-la, Yunnan,China | 4220 | 2020 |  |
| *Rheum alexandrae* | 99.92171 | 28.16012 | Shangri-la, Yunnan,China | 4295 | 2020 |  |
