# Supplementary material for: Global warming pushes the distribution range of the two alpine ‘glasshouse’ Rheum species north- and upwards in the Eastern Himalayas and the Hengduan Mountains
Source: Front Plant Sci. 2022 Oct 7;13:925296. doi: 10.3389/fpls.2022.925296 (PMC9585287; doi:10.3389/fpls.2022.925296)
Supplement: Supplementary file 7 [file Table_2.docx]

**Supplementary Table S2 |** List of General Circulation Models used to derive multi-model median (MMM) GCMs for last glacial maximum (LGM; ca. 22 Kya) and future (2070; RCP 4.5) scenarios.

| General Circulation Models (GCMs) | Code | LGM | Future (2070; RCP 4.5) |
| --- | --- | --- | --- |
| ACCESS1-0 | AC | ˟ | √ |
| BCC-CSM1-1 | BC | ˟ | √ |
| CCSM4 | CC | √ | √ |
| CESM1-CAM5-1-FV2 | CE | ˟ | √ |
| CNRM-CM5 | CN | ˟ | √ |
| GFDL-CM3 | GF | ˟ | √ |
| GFDL-ESM2G | GD | ˟ | √ |
| GISS-E2-R | GS | ˟ | √ |
| HadGEM2-AO | HD | ˟ | √ |
| HadGEM2-CC | HG | ˟ | √ |
| HadGEM2-ES | HE | ˟ | √ |
| INMCM4 | IN | ˟ | √ |
| IPSL-CM5A-LR | IP | ˟ | √ |
| MIROC-ESM-CHEM | MI | ˟ | √ |
| MIROC-ESM | MR | √ | √ |
| MIROC5 | MC | ˟ | √ |
| MPI-ESM-LR | MP | ˟ | √ |
| MPI-ESM-P | ME | √ | ˟ |
| MRI-CGCM3 | MG | ˟ | √ |
| NorESM1-M | NO | ˟ | √ |

˟, GCMs not used; and √, GCMs used in the deriving multi-model median GCMs.

Refer to http://www.worldclim.org for general circulation models (GCMs).
