# Supplementary material for: Global warming pushes the distribution range of the two alpine ‘glasshouse’ Rheum species north- and upwards in the Eastern Himalayas and the Hengduan Mountains
Source: Front Plant Sci. 2022 Oct 7;13:925296. doi: 10.3389/fpls.2022.925296 (PMC9585287; doi:10.3389/fpls.2022.925296)
Supplement: Supplementary file 8 [file Table_3.docx]

**Supplementary Table S3 |** Categories-wise consensus land-cover selection for *Rheum nobile* and *Rheum alexandrae* based on the presence of the number of occurrence records.

| **Consensus Landcover (*V6*)** | **Number of occurrence points** | |
| --- | --- | --- |
|  | *Rheum nobile* | *Rheum alexandrae* |
| Evergreen/deciduous needleleaf trees (lulc1) | 37 | 34 |
| Evergreen broadleaf trees (lulc2) | 9 | 7 |
| Deciduous broadleaf trees (lulc3) | 7 | 2 |
| Mixed/other trees (lulc4) | 34 | 25 |
| Shrubs (lulc5) | 19 | 4 |
| Herbaceous vegetation (lulc6) | 48 | 49 |
| Cultivated and managed vegetation (lulc7) | 35 | 42 |
| Regularly flooded vegetation (lulc8) | 0 | 0 |
| Urban/built-up (lulc9) | 0 | 0 |
| Snow/ice (lulc10) | 17 | 3 |
| Barren (lulc11) | 6 | 1 |
| Open water (lulc12) | 5 | 2 |
| **30% threshold (of total occurrence points)** | 17 (56) | 16 (54) |
