# Supplementary material for: Global warming pushes the distribution range of the two alpine ‘glasshouse’ Rheum species north- and upwards in the Eastern Himalayas and the Hengduan Mountains
Source: Front Plant Sci. 2022 Oct 7;13:925296. doi: 10.3389/fpls.2022.925296 (PMC9585287; doi:10.3389/fpls.2022.925296)
Supplement: Supplementary file 9 [file Table_4.docx]

**Supplementary Table S4 |** Categories-wise Pearson correlation (r) analysis for the selection of explanatory variables for *Rheum nobile*.

| ***V1*** | bio1 | bio2 | **bio3** | bio4 | bio5 | bio6 | **bio7** | bio8 | bio9 | **bio10** | bio11 | bio12 | **bio13** | bio14 | **bio15** | **bio16** | **bio17** | bio18 | **bio19** |
| --- | --- | --- | --- | --- | --- | --- | --- | --- | --- | --- | --- | --- | --- | --- | --- | --- | --- | --- | --- |
| bio1 | 1.00 |  |  |  |  |  |  |  |  |  |  |  |  |  |  |  |  |  |  |
| bio2 | -0.02 | 1.00 |  |  |  |  |  |  |  |  |  |  |  |  |  |  |  |  |  |
| **bio3** | 0.27 | 0.84 | 1.00 |  |  |  |  |  |  |  |  |  |  |  |  |  |  |  |  |
| bio4 | -0.63 | 0.00 | -0.53 | 1.00 |  |  |  |  |  |  |  |  |  |  |  |  |  |  |  |
| bio5 | 0.99 | 0.03 | 0.24 | -0.52 | 1.00 |  |  |  |  |  |  |  |  |  |  |  |  |  |  |
| bio6 | 0.98 | -0.15 | 0.20 | -0.71 | 0.95 | 1.00 |  |  |  |  |  |  |  |  |  |  |  |  |  |
| **bio7** | -0.45 | 0.54 | -0.01 | 0.83 | -0.32 | -0.61 | 1.00 |  |  |  |  |  |  |  |  |  |  |  |  |
| bio8 | 0.99 | 0.00 | 0.21 | -0.53 | 0.99 | 0.95 | -0.35 | 1.00 |  |  |  |  |  |  |  |  |  |  |  |
| bio9 | 0.97 | -0.05 | 0.26 | -0.64 | 0.94 | 0.95 | -0.49 | 0.95 | 1.00 |  |  |  |  |  |  |  |  |  |  |
| **bio10** | 0.95 | -0.12 | 0.11 | -0.51 | 0.95 | 0.93 | -0.40 | 0.96 | 0.97 | 1.00 |  |  |  |  |  |  |  |  |  |
| bio11 | 0.99 | -0.03 | 0.30 | -0.70 | 0.97 | 0.99 | -0.52 | 0.97 | 0.97 | 0.93 | 1.00 |  |  |  |  |  |  |  |  |
| bio12 | 0.02 | -0.24 | -0.03 | -0.29 | -0.07 | 0.07 | -0.40 | -0.01 | 0.17 | 0.03 | 0.07 | 1.00 |  |  |  |  |  |  |  |
| **bio13** | 0.01 | -0.13 | 0.06 | -0.28 | -0.08 | 0.04 | -0.33 | -0.02 | 0.13 | -0.02 | 0.05 | 0.97 | 1.00 |  |  |  |  |  |  |
| bio14 | 0.12 | -0.33 | -0.08 | -0.36 | 0.03 | 0.18 | -0.48 | 0.08 | 0.25 | 0.14 | 0.16 | 0.85 | 0.73 | 1.00 |  |  |  |  |  |
| **bio15** | -0.19 | 0.59 | 0.37 | 0.24 | -0.13 | -0.27 | 0.50 | -0.17 | -0.30 | -0.31 | -0.22 | -0.35 | -0.14 | -0.55 | 1.00 |  |  |  |  |
| **bio16** | 0.79 | 0.15 | 0.43 | -0.63 | 0.76 | 0.77 | -0.39 | 0.75 | 0.79 | 0.68 | 0.80 | 0.13 | 0.14 | 0.14 | -0.05 | 1.00 |  |  |  |
| **bio17** | 0.54 | 0.15 | 0.49 | -0.67 | 0.48 | 0.56 | -0.46 | 0.48 | 0.59 | 0.40 | 0.58 | 0.37 | 0.34 | 0.43 | -0.20 | 0.79 | 1.00 |  |  |
| bio18 | 0.79 | 0.15 | 0.43 | -0.64 | 0.76 | 0.77 | -0.39 | 0.75 | 0.79 | 0.68 | 0.80 | 0.13 | 0.14 | 0.14 | -0.05 | 1.00 | 0.79 | 1.00 |  |
| **bio19** | 0.02 | -0.23 | -0.05 | -0.26 | -0.06 | 0.06 | -0.35 | 0.00 | 0.16 | 0.04 | 0.06 | 0.87 | 0.77 | 0.94 | -0.49 | 0.07 | 0.34 | 0.07 | 1.00 |
| ***V2*** | **ele** | **annSR** | **annRH** | **annWV** | **ai** | **asp** | **pet** | **npp** | **soil_pH** | **soilM** | **soilC** |  |  |  |  |  |  |  |  |
| **ele** | 1.00 |  |  |  |  |  |  |  |  |  |  |  |  |  |  |  |  |  |  |
| **annSR** | -0.07 | 1.00 |  |  |  |  |  |  |  |  |  |  |  |  |  |  |  |  |  |
| **annRH** | -0.67 | 0.34 | 1.00 |  |  |  |  |  |  |  |  |  |  |  |  |  |  |  |  |
| **annWV** | -0.97 | 0.08 | 0.64 | 1.00 |  |  |  |  |  |  |  |  |  |  |  |  |  |  |  |
| **ai** | -0.64 | -0.27 | 0.30 | 0.75 | 1.00 |  |  |  |  |  |  |  |  |  |  |  |  |  |  |
| **asp** | -0.14 | -0.07 | 0.04 | 0.22 | 0.22 | 1.00 |  |  |  |  |  |  |  |  |  |  |  |  |  |
| **pet** | -0.37 | -0.23 | 0.32 | 0.44 | 0.65 | 0.11 | 1.00 |  |  |  |  |  |  |  |  |  |  |  |  |
| **npp** | -0.38 | 0.07 | 0.58 | 0.32 | 0.17 | -0.24 | 0.22 | 1.00 |  |  |  |  |  |  |  |  |  |  |  |
| **soil_pH** | -0.01 | -0.78 | -0.29 | 0.01 | 0.16 | 0.18 | 0.18 | -0.20 | 1.00 |  |  |  |  |  |  |  |  |  |  |
| **soilM** | -0.13 | 0.27 | 0.27 | 0.03 | -0.29 | -0.20 | -0.49 | 0.34 | -0.14 | 1.00 |  |  |  |  |  |  |  |  |  |
| **soilC** | 0.35 | 0.65 | -0.10 | -0.29 | -0.27 | -0.13 | -0.07 | -0.13 | -0.76 | -0.21 | 1.00 |  |  |  |  |  |  |  |  |
| ***V3*** | **cont** | **corr** | **cv** | diss | ent | **even** | **homo** | **max** | **range** | shan | simp | std | uni | var |  |  |  |  |  |
| **cont** | 1.00 |  |  |  |  |  |  |  |  |  |  |  |  |  |  |  |  |  |  |
| **corr** | 0.30 | 1.00 |  |  |  |  |  |  |  |  |  |  |  |  |  |  |  |  |  |
| **cv** | 0.61 | 0.69 | 1.00 |  |  |  |  |  |  |  |  |  |  |  |  |  |  |  |  |
| diss | 0.92 | 0.42 | 0.64 | 1.00 |  |  |  |  |  |  |  |  |  |  |  |  |  |  |  |
| ent | 0.65 | 0.61 | 0.55 | 0.87 | 1.00 |  |  |  |  |  |  |  |  |  |  |  |  |  |  |
| **even** | 0.50 | 0.68 | 0.52 | 0.67 | 0.82 | 1.00 |  |  |  |  |  |  |  |  |  |  |  |  |  |
| **homo** | -0.64 | -0.43 | -0.45 | -0.87 | -0.96 | -0.72 | 1.00 |  |  |  |  |  |  |  |  |  |  |  |  |
| **max** | -0.04 | -0.33 | -0.08 | -0.39 | -0.72 | -0.65 | 0.72 | 1.00 |  |  |  |  |  |  |  |  |  |  |  |
| **range** | 0.77 | 0.59 | 0.71 | 0.88 | 0.86 | 0.60 | -0.79 | -0.42 | 1.00 |  |  |  |  |  |  |  |  |  |  |
| shan | 0.72 | 0.72 | 0.70 | 0.89 | 0.97 | 0.82 | -0.89 | -0.59 | 0.92 | 1.00 |  |  |  |  |  |  |  |  |  |
| simp | 0.60 | 0.70 | 0.59 | 0.81 | 0.98 | 0.84 | -0.91 | -0.70 | 0.84 | 0.97 | 1.00 |  |  |  |  |  |  |  |  |
| std | 0.82 | 0.71 | 0.83 | 0.90 | 0.84 | 0.73 | -0.76 | -0.35 | 0.93 | 0.94 | 0.85 | 1.00 |  |  |  |  |  |  |  |
| uni | -0.42 | -0.48 | -0.35 | -0.68 | -0.93 | -0.74 | 0.92 | 0.84 | -0.69 | -0.84 | -0.93 | -0.65 | 1.00 |  |  |  |  |  |  |
| var | 0.86 | 0.62 | 0.82 | 0.86 | 0.72 | 0.65 | -0.65 | -0.20 | 0.87 | 0.85 | 0.72 | 0.97 | -0.51 | 1.00 |  |  |  |  |  |
| ***V4*** | **gdd** | fgd | **gsl** | **gst** | lgd |  |  |  |  |  |  |  |  |  |  |  |  |  |  |
| **gdd** | 1.00 |  |  |  |  |  |  |  |  |  |  |  |  |  |  |  |  |  |  |
| fgd | -0.22 | 1.00 |  |  |  |  |  |  |  |  |  |  |  |  |  |  |  |  |  |
| **gsl** | 0.60 | 0.04 | 1.00 |  |  |  |  |  |  |  |  |  |  |  |  |  |  |  |  |
| **gst** | 0.02 | 0.84 | 0.38 | 1.00 |  |  |  |  |  |  |  |  |  |  |  |  |  |  |  |
| lgd | -0.22 | 1.00 | 0.04 | 0.84 | 1.00 |  |  |  |  |  |  |  |  |  |  |  |  |  |  |
| ***V5*** | uvb1 | **uvb2** | uvb3 | **uvb4** | uvb5 | uvb6 |  |  |  |  |  |  |  |  |  |  |  |  |  |
| uvb1 | 1.00 |  |  |  |  |  |  |  |  |  |  |  |  |  |  |  |  |  |  |
| **uvb2** | 0.96 | 1.00 |  |  |  |  |  |  |  |  |  |  |  |  |  |  |  |  |  |
| uvb3 | 0.99 | 0.98 | 1.00 |  |  |  |  |  |  |  |  |  |  |  |  |  |  |  |  |
| **uvb4** | 0.83 | 0.63 | 0.77 | 1.00 |  |  |  |  |  |  |  |  |  |  |  |  |  |  |  |
| uvb5 | 0.99 | 0.98 | 1.00 | 0.75 | 1.00 |  |  |  |  |  |  |  |  |  |  |  |  |  |  |
| uvb6 | 0.94 | 0.80 | 0.90 | 0.96 | 0.89 | 1.00 |  |  |  |  |  |  |  |  |  |  |  |  |  |
| ***V6*** | **lulc1** | **lulc4** | **lulc5** | **lulc6** | **lulc7** | **lulc10** |  |  |  |  |  |  |  |  |  |  |  |  |  |
| **lulc1** | 1.00 |  |  |  |  |  |  |  |  |  |  |  |  |  |  |  |  |  |  |
| **lulc4** | 0.23 | 1.00 |  |  |  |  |  |  |  |  |  |  |  |  |  |  |  |  |  |
| **lulc5** | -0.23 | -0.16 | 1.00 |  |  |  |  |  |  |  |  |  |  |  |  |  |  |  |  |
| **lulc6** | -0.47 | -0.57 | -0.25 | 1.00 |  |  |  |  |  |  |  |  |  |  |  |  |  |  |  |
| **lulc7** | -0.28 | -0.12 | -0.14 | 0.15 | 1.00 |  |  |  |  |  |  |  |  |  |  |  |  |  |  |
| **lulc10** | -0.33 | -0.40 | 0.11 | 0.20 | -0.43 | 1.00 |  |  |  |  |  |  |  |  |  |  |  |  |  |

|r| < 0.8 are considered as least correlated variables (bold text) and are considered in the analysis.

***V1***, Bioclimatic variables; ***V2***, geo-climatic variables; ***V3***, Habitat heterogeneity; ***V4***, Growing days; ***V5***, Ultra-violet radiations; ***V6***, Consensus land-cover

Refer to Table 1 for the bioclimatic variables.
