# Supplementary material for: Global warming pushes the distribution range of the two alpine ‘glasshouse’ Rheum species north- and upwards in the Eastern Himalayas and the Hengduan Mountains
Source: Front Plant Sci. 2022 Oct 7;13:925296. doi: 10.3389/fpls.2022.925296 (PMC9585287; doi:10.3389/fpls.2022.925296)
Supplement: Supplementary file 11 [file Table_6.docx]

**Supplementary Table S6 |** Categories-wise Variance Inflation Factor (VIF) in different test runs for the selection of explanatory variables (VIF<10, bold text) for *Rheum nobile*.

| ***V1*** | Run1 | Run2 | Run3 | Run4 | Run5 | Run6 | Run7 | Run8 | Run9 | Run10 | Run11 | Run12 |
| --- | --- | --- | --- | --- | --- | --- | --- | --- | --- | --- | --- | --- |
| **bio7** | 1356.67 | 1330.75 | 307.79 | 306.83 | 50.40 | 44.44 | 44.39 | 43.31 | 40.11 | 2.04 | 2.04 | 1.87 |
| **bio3** | 826.36 | 825.92 | 714.29 | 684.09 | 22.41 | 21.54 | 21.47 | 19.95 | 18.62 | 2.12 | 2.08 | 1.93 |
| **bio10** | 186.68 | 186.68 | 186.28 | 139.28 | 129.30 | 127.68 | 121.35 | 22.37 | 19.88 | 19.66 | 2.93 | 2.83 |
| **bio15** | 13.66 | 13.61 | 13.54 | 13.53 | 11.87 | 9.40 | 3.94 | 3.86 | 3.84 | 3.84 | 3.12 | 3.12 |
| **bio13** | 177.86 | 177.41 | 176.42 | 170.48 | 158.36 | 148.00 | 5.61 | 4.18 | 3.87 | 3.70 | 3.47 | 3.46 |
| **bio19** | 18.48 | 17.93 | 17.85 | 13.72 | 13.52 | 13.50 | 12.96 | 12.73 | 12.55 | 12.50 | 12.20 | 4.29 |
| **bio17** | 13.99 | 13.87 | 13.82 | 13.81 | 10.47 | 10.07 | 9.73 | 6.99 | 6.98 | 6.46 | 5.90 | 5.18 |
| **bio16** | 208799.61 | 8.71 | 8.70 | 8.68 | 7.94 | 7.54 | 7.53 | 7.35 | 7.31 | 6.77 | 6.68 | 6.33 |
| bio14 | 30.25 | 29.94 | 29.89 | 17.88 | 16.26 | 16.18 | 13.51 | 13.22 | 12.89 | 12.58 | 12.30 |  |
| bio5 | 8706.87 | 8633.13 | 842.94 | 323.93 | 322.32 | 94.11 | 92.83 | 91.80 | 22.36 | 20.50 |  |  |
| bio4 | 109.92 | 108.14 | 100.85 | 89.86 | 84.74 | 70.02 | 69.96 | 69.50 | 64.12 |  |  |  |
| bio8 | 176.26 | 175.02 | 171.75 | 171.55 | 160.95 | 105.45 | 101.75 | 101.27 |  |  |  |  |
| bio9 | 235.62 | 235.00 | 234.98 | 189.98 | 179.49 | 179.14 | 174.13 |  |  |  |  |  |
| bio12 | 224.57 | 223.53 | 222.16 | 218.65 | 208.80 | 200.04 |  |  |  |  |  |  |
| bio11 | 2964.08 | 2562.68 | 2562.66 | 713.65 | 676.48 |  |  |  |  |  |  |  |
| bio2 | 1054.84 | 1053.48 | 933.31 | 911.70 |  |  |  |  |  |  |  |  |
| bio1 | 4823.52 | 4140.55 | 4140.04 |  |  |  |  |  |  |  |  |  |
| bio6 | 12018.89 | 11586.60 |  |  |  |  |  |  |  |  |  |  |
| bio18 | 209129.68 |  |  |  |  |  |  |  |  |  |  |  |
| ***V2*** | Run1 | Run2 |  |  |  |  |  |  |  |  |  |  |
| **asp** | 1.3 | 1.25 |  |  |  |  |  |  |  |  |  |  |
| **npp** | 2.1 | 2.1 |  |  |  |  |  |  |  |  |  |  |
| **soilM** | 2.73 | 2.54 |  |  |  |  |  |  |  |  |  |  |
| **ai** | 7.08 | 2.65 |  |  |  |  |  |  |  |  |  |  |
| **pet** | 3.65 | 3.48 |  |  |  |  |  |  |  |  |  |  |
| **annRH** | 4.03 | 4 |  |  |  |  |  |  |  |  |  |  |
| **annSR** | 5.35 | 5.31 |  |  |  |  |  |  |  |  |  |  |
| **soil_pH** | 5.91 | 5.89 |  |  |  |  |  |  |  |  |  |  |
| **soilC** | 6 | 5.94 |  |  |  |  |  |  |  |  |  |  |
| annWV | 15.02 |  |  |  |  |  |  |  |  |  |  |  |
| ***V3*** | Run1 | Run2 | Run3 | Run4 | Run5 | Run6 | Run7 | Run8 | Run9 |  |  |  |
| **corr** | 15.65 | 14.34 | 12.51 | 11.04 | 10.95 | 5.99 | 3.61 | 3.26 | 3.18 |  |  |  |
| **max** | 21.26 | 21.02 | 19.25 | 15.68 | 15.68 | 15.03 | 5.67 | 4.8 | 3.32 |  |  |  |
| **cv** | 8.52 | 7.49 | 5.34 | 4.95 | 4.66 | 4.28 | 4.01 | 3.95 | 3.43 |  |  |  |
| **even** | 16.46 | 16.46 | 16.28 | 16.19 | 14.69 | 9.19 | 5.8 | 5.79 | 3.85 |  |  |  |
| **range** | 32.46 | 29.69 | 27.02 | 26.71 | 21.96 | 12.39 | 9.42 | 9.32 | 4.87 |  |  |  |
| **homo** | 99.53 | 96.94 | 96.14 | 60.58 | 20.38 | 19.91 | 12.79 | 6.58 | 6.55 |  |  |  |
| var | 218.56 | 217.51 | 21.34 | 20.9 | 20.19 | 14.27 | 11.2 | 10.98 |  |  |  |  |
| uni | 108.11 | 99.37 | 95.55 | 53.47 | 52.15 | 13.09 | 12.87 |  |  |  |  |  |
| cont | 97.34 | 90.45 | 85.73 | 23.14 | 23.13 | 21.15 |  |  |  |  |  |  |
| simp | 239.73 | 194.01 | 189.57 | 188.42 | 133.31 |  |  |  |  |  |  |  |
| ent | 484.38 | 289.32 | 250.78 | 212.51 |  |  |  |  |  |  |  |  |
| diss | 258.52 | 253.35 | 251.59 |  |  |  |  |  |  |  |  |  |
| std | 622.91 | 502.99 |  |  |  |  |  |  |  |  |  |  |
| shan | 736.76 |  |  |  |  |  |  |  |  |  |  |  |
| ***V4*** | Run1 | Run2 |  |  |  |  |  |  |  |  |  |  |
| **gdd** | 1.72 | 1.71 |  |  |  |  |  |  |  |  |  |  |
| **gsl** | 2.39 | 2.38 |  |  |  |  |  |  |  |  |  |  |
| **lgd** | 7314.17 | 5.04 |  |  |  |  |  |  |  |  |  |  |
| **gst** | 6.11 | 5.82 |  |  |  |  |  |  |  |  |  |  |
| fgd | 7391.42 |  |  |  |  |  |  |  |  |  |  |  |
| ***V5*** | Run1 | Run2 | Run3 | Run4 | Run5 |  |  |  |  |  |  |  |
| **uvb2** | 2842.56 | 1218.56 | 350.65 | 13.5 | 1.65 |  |  |  |  |  |  |  |
| **uvb4** | 185.66 | 127.4 | 105.81 | 69.22 | 1.65 |  |  |  |  |  |  |  |
| uvb6 | 425.58 | 395.64 | 115.97 | 115.93 |  |  |  |  |  |  |  |  |
| uvb3 | 949.26 | 525.94 | 505.5 |  |  |  |  |  |  |  |  |  |
| uvb1 | 3545.77 | 1869.89 |  |  |  |  |  |  |  |  |  |  |
| uvb5 | 3678.44 |  |  |  |  |  |  |  |  |  |  |  |
| ***V6*** | Run1 |  |  |  |  |  |  |  |  |  |  |  |
| **lulc5** | 2.06 |  |  |  |  |  |  |  |  |  |  |  |
| **lulc7** | 2.47 |  |  |  |  |  |  |  |  |  |  |  |
| **lulc10** | 2.81 |  |  |  |  |  |  |  |  |  |  |  |
| **lulc1** | 3.08 |  |  |  |  |  |  |  |  |  |  |  |
| **lulc4** | 3.1 |  |  |  |  |  |  |  |  |  |  |  |
| **lulc6** | 3.36 |  |  |  |  |  |  |  |  |  |  |  |

***V1***, Bioclimatic variables; ***V2***, geo-climatic variables; ***V3***, Habitat heterogeneity; ***V4***, Growing days; ***V5***, Ultra-violet radiations; ***V6***, Consensus land-cover

Refer to Table 1 for the bioclimatic variables.
