# Supplementary material for: Global warming pushes the distribution range of the two alpine ‘glasshouse’ Rheum species north- and upwards in the Eastern Himalayas and the Hengduan Mountains
Source: Front Plant Sci. 2022 Oct 7;13:925296. doi: 10.3389/fpls.2022.925296 (PMC9585287; doi:10.3389/fpls.2022.925296)
Supplement: Supplementary file 12 [file Table_7.docx]

**Supplementary Table S7 |** Categories-wise Variance Inflation Factor (VIF) in different test runs for the selection of explanatory variables (VIF<10, bold text) for *Rheum alexandrae*.

| ***V1*** | Run1 | Run2 | Run3 | Run4 | Run5 | Run6 | Run7 | Run8 | Run9 | Run10 |
| --- | --- | --- | --- | --- | --- | --- | --- | --- | --- | --- |
| **bio13** | 44.61 | 44.6 | 44.35 | 39.55 | 35.93 | 34.08 | 29.91 | 3.17 | 2.97 | 2.86 |
| **bio3** | 120.69 | 120.68 | 118.83 | 118.73 | 77.67 | 4.68 | 4.55 | 4.11 | 3.53 | 3.49 |
| **bio2** | 342.09 | 341.13 | 330.4 | 325.98 | 105.18 | 5.75 | 5.15 | 4.33 | 4.09 | 3.85 |
| **bio19** | 92.49 | 91.16 | 90.98 | 89.99 | 88.88 | 75.81 | 11.31 | 5.53 | 5.53 | 4.47 |
| **bio16** | 19.74 | 18.94 | 18.88 | 15.2 | 13.27 | 7.33 | 6.9 | 5.19 | 5.04 | 4.95 |
| **bio15** | 16.59 | 16.58 | 16.49 | 16.36 | 15.53 | 15.19 | 12.28 | 6.42 | 6.28 | 6.12 |
| **bio17** | 24.91 | 23.52 | 23.16 | 22.63 | 18.59 | 12.88 | 10.66 | 9.97 | 8.07 | 7.26 |
| **bio10** | 84.53 | 81.41 | 81.4 | 78.51 | 78.49 | 46.22 | 44.91 | 40.01 | 13.05 | 7.7 |
| **bio8** | 31 | 28.3 | 27.65 | 22.45 | 18.92 | 18.63 | 18.27 | 18.06 | 14.05 | 9.35 |
| bio5 | 1586.44 | 1578.98 | 1116.86 | 34.37 | 30.48 | 30.44 | 27.85 | 25.08 | 23.8 |  |
| bio9 | 82.83 | 82.23 | 81.57 | 81.5 | 81.49 | 44.74 | 44.74 | 44.2 |  |  |
| bio12 | 75.41 | 75.4 | 75.36 | 61.21 | 55.2 | 52 | 47.21 |  |  |  |
| bio14 | 112.29 | 111.5 | 111.48 | 106.81 | 106.08 | 91.76 |  |  |  |  |
| bio4 | 403.92 | 248.69 | 243.94 | 198.53 | 148.32 |  |  |  |  |  |
| bio7 | 1969.13 | 1951.15 | 596.56 | 542.61 |  |  |  |  |  |  |
| bio1 | 4203.63 | 1890.48 | 1650.01 |  |  |  |  |  |  |  |
| bio6 | 2825.8 | 2758.02 |  |  |  |  |  |  |  |  |
| bio11 | 4259.19 |  |  |  |  |  |  |  |  |  |
| bio18 | alias in the model | | |  |  |  |  |  |  |  |
| ***V2*** | Run1 |  |  |  |  |  |  |  |  |  |
| **asp** | 1.26 |  |  |  |  |  |  |  |  |  |
| **npp** | 1.68 |  |  |  |  |  |  |  |  |  |
| **annSR** | 3.01 |  |  |  |  |  |  |  |  |  |
| **soilM** | 4.26 |  |  |  |  |  |  |  |  |  |
| **annRH** | 3.30 |  |  |  |  |  |  |  |  |  |
| **soil_pH** | 4.33 |  |  |  |  |  |  |  |  |  |
| **pet** | 6.70 |  |  |  |  |  |  |  |  |  |
| **soilC** | 7.59 |  |  |  |  |  |  |  |  |  |
| **ai** | 2.28 |  |  |  |  |  |  |  |  |  |
| **annWV** | 3.31 |  |  |  |  |  |  |  |  |  |
| ***V3*** | Run1 | Run2 | Run3 | Run4 | Run5 | Run6 | Run7 | Run8 | Run9 |  |
| **even** | 17.30 | 16.39 | 12.43 | 12.12 | 11.52 | 8.46 | 4.95 | 2.25 | 2.25 |  |
| **corr** | 18.04 | 13.95 | 12.81 | 12.36 | 12.32 | 7.72 | 7.70 | 4.34 | 3.33 |  |
| **count** | 168.39 | 164.67 | 12.43 | 9.65 | 8.82 | 7.88 | 7.64 | 6.16 | 5.09 |  |
| **cv** | 10.77 | 8.62 | 8.56 | 8.52 | 8.51 | 8.51 | 8.50 | 8.02 | 5.26 |  |
| **max** | 8.43 | 8.18 | 8.16 | 8.13 | 8.09 | 7.81 | 6.90 | 6.84 | 6.82 |  |
| **homo** | 75.24 | 75.21 | 34.68 | 31.92 | 15.65 | 14.45 | 9.80 | 8.48 | 8.29 |  |
| var | 220.22 | 22.04 | 21.47 | 15.14 | 14.68 | 14.15 | 12.84 | 12.64 |  |  |
| range | 32.21 | 30.85 | 27.58 | 25.24 | 25.21 | 19.50 | 14.95 |  |  |  |
| simp | 322.83 | 310.39 | 305.97 | 143.93 | 107.03 | 23.54 |  |  |  |  |
| uni | 193.34 | 179.17 | 151.36 | 73.69 | 72.57 |  |  |  |  |  |
| ent | 489.60 | 488.34 | 290.73 | 167.72 |  |  |  |  |  |  |
| shan | 458.81 | 377.40 | 363.11 |  |  |  |  |  |  |  |
| diss | 511.65 | 508.99 |  |  |  |  |  |  |  |  |
| std | 605.78 |  |  |  |  |  |  |  |  |  |
| ***V4*** | Run1 | Run2 |  |  |  |  |  |  |  |  |
| **gdd** | 1.11 | 1.11 |  |  |  |  |  |  |  |  |
| **gsl** | 16.29 | 1.94 |  |  |  |  |  |  |  |  |
| **lgd** | 8.14 | 3.21 |  |  |  |  |  |  |  |  |
| **gst** | 5.87 | 3.78 |  |  |  |  |  |  |  |  |
| fgd | 28.97 |  |  |  |  |  |  |  |  |  |
| ***V5*** | Run1 | Run2 | Run3 | Run4 | Run5 |  |  |  |  |  |
| **uvb2** | 652.59 | 615.33 | 38.54 | 22.26 | 1.01 |  |  |  |  |  |
| **uvb6** | 1033.64 | 878.40 | 276.94 | 67.11 | 1.01 |  |  |  |  |  |
| uvb3 | 344.95 | 220.28 | 118.82 | 93.93 |  |  |  |  |  |  |
| uvb4 | 667.50 | 547.99 | 348.05 |  |  |  |  |  |  |  |
| uvb5 | 2323.70 | 1318.92 |  |  |  |  |  |  |  |  |
| uvb1 | 3818.55 |  |  |  |  |  |  |  |  |  |
| ***V6*** | Run1 |  |  |  |  |  |  |  |  |  |
| **lulc1** | 3.26 |  |  |  |  |  |  |  |  |  |
| **lulc4** | 2.94 |  |  |  |  |  |  |  |  |  |
| **lulc6** | 3.68 |  |  |  |  |  |  |  |  |  |
| **lulc7** | 2.64 |  |  |  |  |  |  |  |  |  |

***V1***, Bioclimatic variables; ***V2***, geo-climatic variables; ***V3***, Habitat heterogeneity; ***V4***, Growing days; ***V5***, Ultra-violet radiations; ***V6***, Consensus land-cover

Refer to Table 1 for the bioclimatic variables.
