# Supplementary material for: Global warming pushes the distribution range of the two alpine ‘glasshouse’ Rheum species north- and upwards in the Eastern Himalayas and the Hengduan Mountains
Source: Front Plant Sci. 2022 Oct 7;13:925296. doi: 10.3389/fpls.2022.925296 (PMC9585287; doi:10.3389/fpls.2022.925296)
Supplement: Supplementary file 13 [file Table_8.docx]

**Supplementary Table S8 |** Variance Inflation Factor (VIF) in different test runs for the selection of explanatory variables combining all categories of variables (VIF<10, bold text) for *Rheum nobile.*

| **Variables** | Run1 | Run2 | Run3 | Run4 | Run5 | Run6 | Run7 | Run8 | Run9 |
| --- | --- | --- | --- | --- | --- | --- | --- | --- | --- |
| **gst** | 4.26 | 3.99 | 3.29 | 3.23 | 2.67 | 2.57 | 2.55 | 2.44 | 2.38 |
| **asp** | 3.77 | 3.77 | 3.76 | 3.73 | 3.16 | 2.84 | 2.83 | 2.75 | 2.44 |
| **max** | 10.34 | 10.34 | 10.28 | 9.2 | 5.48 | 5.39 | 5.13 | 3.86 | 3.17 |
| **range** | 15.12 | 15 | 14.96 | 14.92 | 8.07 | 7.85 | 7.83 | 3.82 | 3.51 |
| **corr** | 7.27 | 7.22 | 7.2 | 7.1 | 5.64 | 5.6 | 5.6 | 4.41 | 3.86 |
| **bio3** | 31.92 | 31.27 | 28.8 | 7.4 | 7.37 | 6.93 | 5.22 | 4.22 | 4.21 |
| **soilM** | 8.64 | 8.63 | 8.61 | 7.94 | 7.88 | 7.85 | 6.09 | 4.54 | 4.25 |
| **npp** | 7.55 | 7.47 | 7.35 | 7.28 | 7.23 | 7.23 | 6.88 | 6.88 | 4.31 |
| **lulc7** | 6.91 | 6.24 | 5.79 | 5.76 | 5.74 | 4.81 | 4.81 | 4.52 | 4.37 |
| **bio13** | 16.54 | 16.3 | 16.29 | 14.65 | 14.63 | 13.28 | 8.44 | 8.3 | 4.4 |
| **lulc10** | 7.46 | 6.72 | 6.46 | 6.11 | 5.79 | 4.83 | 4.78 | 4.77 | 4.77 |
| **lulc1** | 10.46 | 9.03 | 9.01 | 9 | 8.86 | 7.22 | 6.93 | 5.6 | 5.18 |
| **bio7** | 8.17 | 8.08 | 8.05 | 5.39 | 5.36 | 5.22 | 5.22 | 5.22 | 5.21 |
| **uvb2** | 32.41 | 31.56 | 31.48 | 6.38 | 6.32 | 6.3 | 6.19 | 6.15 | 5.25 |
| **lulc4** | 8.89 | 7.08 | 6.97 | 6.42 | 6.39 | 5.94 | 5.93 | 5.93 | 5.45 |
| **lulc5** | 7.89 | 7.53 | 7.5 | 6.98 | 6.94 | 6.55 | 6.53 | 6.48 | 5.48 |
| **soil_pH** | 25.87 | 24.33 | 22.12 | 16.73 | 16.05 | 7.3 | 6.02 | 5.88 | 5.72 |
| **even** | 7.96 | 7.82 | 7.4 | 7.27 | 6.82 | 6.58 | 6.54 | 6.54 | 5.86 |
| **annRH** | 23.38 | 20.42 | 15.58 | 10.76 | 10.44 | 9.69 | 6.79 | 6.65 | 6.28 |
| **annSR** | 17.27 | 16.97 | 15.09 | 10.08 | 10.07 | 8.67 | 7.24 | 7.18 | 6.78 |
| **pet** | 9.13 | 8.93 | 7.78 | 7.78 | 7.54 | 6.94 | 6.87 | 6.82 | 6.81 |
| **lulc6** | 12.83 | 10.57 | 10.46 | 10.46 | 9.9 | 9.16 | 9.15 | 7.82 | 6.82 |
| **bio17** | 26.7 | 20.01 | 19.23 | 18.79 | 18.37 | 12.62 | 9.24 | 8.9 | 7.17 |
| **bio16** | 88.86 | 23.67 | 19.27 | 18.43 | 18.09 | 9.7 | 9.22 | 9.22 | 7.87 |
| **gsl** | 35.75 | 29.11 | 12.48 | 11.73 | 11.28 | 10.55 | 10.07 | 8.6 | 8.08 |
| **gdd** | 13.61 | 12.16 | 12.15 | 12.08 | 12.08 | 10.16 | 10.16 | 10.01 | 8.78 |
| bio19 | 21.51 | 20.89 | 20.85 | 16.93 | 15.62 | 13.58 | 11.98 | 11.71 |  |
| cv | 20.23 | 20.22 | 19.31 | 17.12 | 14.79 | 14.43 | 14.06 |  |  |
| bio15 | 18.52 | 18.2 | 17.87 | 17.77 | 17.65 | 14.57 |  |  |  |
| soilC | 22.76 | 22.72 | 21.62 | 21.13 | 21.05 |  |  |  |  |
| homo | 25.01 | 25.01 | 24.41 | 22.16 |  |  |  |  |  |
| uvb4 | 36.22 | 35.63 | 35.34 |  |  |  |  |  |  |
| bio10 | 65.3 | 39.38 |  |  |  |  |  |  |  |
| ai | 94.2 |  |  |  |  |  |  |  |  |

Refer to Table 1 for the bioclimatic variables.
