# Supplementary material for: Global warming pushes the distribution range of the two alpine ‘glasshouse’ Rheum species north- and upwards in the Eastern Himalayas and the Hengduan Mountains
Source: Front Plant Sci. 2022 Oct 7;13:925296. doi: 10.3389/fpls.2022.925296 (PMC9585287; doi:10.3389/fpls.2022.925296)
Supplement: Supplementary file 14 [file Table_9.docx]

**Supplementary Table S9 |** Variance Inflation Factor (VIF) in different test runs for the selection of explanatory variables combining all categories of variables (VIF<10, bold text) for *Rheum alexandrae*.

| **Variables** | Run1 | Run2 | Run3 | Run4 | Run5 | Run6 | Run7 | Run8 | Run9 | Run10 | Run11 | Run12 |
| --- | --- | --- | --- | --- | --- | --- | --- | --- | --- | --- | --- | --- |
| **asp** | 4.16 | 4.04 | 3.86 | 3.85 | 3.52 | 3.08 | 3.06 | 2.95 | 2.82 | 2.5 | 2.48 | 2.01 |
| **npp** | 4.56 | 3.22 | 3.2 | 2.76 | 2.4 | 2.37 | 2.27 | 2.27 | 2.24 | 2.17 | 2.17 | 2.12 |
| **soil_pH** | 17.51 | 16.95 | 16.92 | 14.8 | 4.7 | 3.42 | 3.41 | 3.37 | 3.26 | 3.17 | 2.94 | 2.37 |
| **cont** | 15.81 | 14.81 | 14.7 | 14.61 | 14.46 | 14.44 | 14.08 | 13.99 | 10.74 | 3.18 | 3.02 | 2.82 |
| **lgd** | 21.51 | 21.35 | 21.17 | 19.56 | 15.97 | 15.61 | 6.89 | 3.51 | 3.39 | 3.37 | 3.02 | 2.86 |
| **corr** | 9.75 | 8.56 | 8.47 | 8.45 | 7.81 | 7.75 | 7.61 | 7.59 | 6.4 | 3.12 | 2.89 | 2.88 |
| **soilM** | 10.48 | 10.29 | 10.08 | 10 | 9.73 | 9.15 | 9.07 | 9.04 | 9.01 | 9 | 8.84 | 3.02 |
| **bio2** | 50.83 | 39.02 | 10.57 | 10.56 | 10.03 | 5.8 | 3.85 | 3.81 | 3.76 | 3.76 | 3.56 | 3.29 |
| **max** | 15.31 | 13.32 | 12.06 | 11.44 | 10.99 | 10.8 | 10.79 | 10.62 | 5.57 | 3.71 | 3.66 | 3.32 |
| **even** | 5.48 | 5.36 | 4.57 | 4.57 | 3.85 | 3.82 | 3.82 | 3.62 | 3.61 | 3.58 | 3.52 | 3.35 |
| **bio13** | 4.49 | 4.25 | 4.12 | 4.09 | 3.97 | 3.89 | 3.83 | 3.82 | 3.72 | 3.69 | 3.61 | 3.56 |
| **lulc7** | 5.5 | 4.71 | 4.42 | 4.38 | 4.16 | 4.16 | 3.81 | 3.8 | 3.72 | 3.72 | 3.69 | 3.69 |
| **annSR** | 34.49 | 25.02 | 13.56 | 7.9 | 6.1 | 5.54 | 5.51 | 5.07 | 5.07 | 4.81 | 3.87 | 3.86 |
| **gdd** | 7.77 | 7.5 | 6.82 | 6.81 | 6.73 | 5.37 | 5.26 | 5.25 | 5.21 | 4.99 | 4.72 | 4.03 |
| **gsl** | 65.02 | 22.52 | 21.26 | 18.02 | 12.06 | 11.98 | 11.73 | 5.97 | 5.95 | 4.97 | 4.74 | 4.7 |
| **lulc4** | 8.58 | 8.53 | 8.15 | 8.09 | 7.18 | 7.17 | 5.98 | 5.69 | 4.94 | 4.93 | 4.92 | 4.91 |
| **lulc1** | 10.67 | 9.65 | 9.64 | 9.59 | 6.97 | 6.96 | 6.51 | 6.3 | 6.21 | 5.62 | 5.41 | 5.23 |
| **lulc6** | 13.89 | 13.23 | 11.6 | 10.95 | 10.08 | 9.94 | 9.94 | 9.63 | 8.76 | 6.93 | 6.53 | 5.65 |
| **bio15** | 25.7 | 25.7 | 24.46 | 23.94 | 23.47 | 11.9 | 9.73 | 7.17 | 7.02 | 6.73 | 6.65 | 6.59 |
| **bio16** | 72.39 | 23.96 | 18.89 | 15.77 | 15.77 | 15.76 | 9.41 | 9.28 | 8.98 | 8.68 | 6.94 | 6.6 |
| **bio3** | 17.93 | 17.73 | 10.92 | 10.44 | 10.33 | 10.29 | 9.91 | 9.5 | 9.4 | 9.28 | 9.23 | 7.12 |
| **annRH** | 16.5 | 16.25 | 13.87 | 12.9 | 12.73 | 11.95 | 10.93 | 10.86 | 10.85 | 10.33 | 7.85 | 7.6 |
| pet | 16.66 | 15.01 | 14.51 | 13.63 | 12.46 | 11.68 | 11.65 | 11.09 | 11.05 | 10.84 | 10.1 |  |
| uvb2 | 32.99 | 32.34 | 18.18 | 16.11 | 15.09 | 13.55 | 13.36 | 12.74 | 12.59 | 11.2 |  |  |
| cv | 16.4 | 15.67 | 15.23 | 15.22 | 15.18 | 13.51 | 13.21 | 12.9 | 12.77 |  |  |  |
| homo | 18.77 | 18.51 | 17.58 | 15.25 | 15.07 | 14.83 | 14.65 | 14.28 |  |  |  |  |
| annWV | 57.51 | 36.4 | 30.6 | 28.22 | 24.58 | 22.35 | 18.47 |  |  |  |  |  |
| gst | 165.31 | 38.59 | 38.49 | 25.07 | 24.35 | 24.27 |  |  |  |  |  |  |
| bio17 | 60.22 | 36.6 | 36.23 | 25.93 | 25.21 |  |  |  |  |  |  |  |
| soilC | 36.33 | 34.53 | 31.58 | 31.56 |  |  |  |  |  |  |  |  |
| bio8 | 60.42 | 49.48 | 49.43 |  |  |  |  |  |  |  |  |  |
| uvb6 | 85.06 | 77.51 |  |  |  |  |  |  |  |  |  |  |
| ai | 294.63 |  |  |  |  |  |  |  |  |  |  |  |

Refer to Table 1 for the bioclimatic variables.
