# Supplementary material for: Global warming pushes the distribution range of the two alpine ‘glasshouse’ Rheum species north- and upwards in the Eastern Himalayas and the Hengduan Mountains
Source: Front Plant Sci. 2022 Oct 7;13:925296. doi: 10.3389/fpls.2022.925296 (PMC9585287; doi:10.3389/fpls.2022.925296)
Supplement: Supplementary file 15 [file Table_10.docx]

**Supplementary Table S10 |** Variance Inflation Factor (VIF) in different test runs for the selection of explanatory variables among bioclimatic variables + each environmental variables categories (VIF<10, bold text) for the two alpine ‘glasshouse’ herbs *Rheum nobile* and *Rheum alexandrae*.

| *R. nobile* | | | | *R. alexandrae* | | | | | |
| --- | --- | --- | --- | --- | --- | --- | --- | --- | --- |
| ***V1* and *V2*** | Run1 | Run2 | Run3 | ***V1* and *V2*** | Run1 | Run2 | Run3 | Run4 | Run5 |
| **asp** | 1.58 | 1.4 | 1.38 | **asp** | 1.75 | 1.53 | 1.43 | 1.43 | 1.38 |
| **bio3** | 4.82 | 4.1 | 1.88 | **soil_pH** | 6.58 | 5.74 | 5.18 | 4.97 | 1.7 |
| **soilM** | 4.42 | 4.07 | 3.52 | **npp** | 2.16 | 1.94 | 1.94 | 1.91 | 1.74 |
| **bio7** | 4.01 | 3.62 | 3.62 | **bio2** | 4.7 | 4.7 | 2.82 | 2.75 | 2.69 |
| **pet** | 4.32 | 4.16 | 4.16 | **bio13** | 3.31 | 3.04 | 3.04 | 3.03 | 3.03 |
| **annRH** | 8.59 | 8.44 | 4.4 | **annSR** | 5.76 | 5.76 | 3.76 | 3.73 | 3.13 |
| **npp** | 4.9 | 4.86 | 4.53 | **bio8** | 16.06 | 11.44 | 9.54 | 3.74 | 3.65 |
| **bio10** | 30.61 | 6.31 | 4.75 | **soilM** | 6.39 | 6.2 | 5.55 | 5.1 | 4.97 |
| **bio13** | 9.47 | 7.2 | 5.1 | **bio15** | 10.93 | 10.79 | 5.83 | 5.16 | 5.1 |
| **annSR** | 7.95 | 7.21 | 5.19 | **annRH** | 6.73 | 6.22 | 6.21 | 5.58 | 5.18 |
| **bio19** | 8.66 | 6.81 | 5.95 | **bio3** | 7.51 | 5.67 | 5.59 | 5.48 | 5.21 |
| **bio17** | 20.86 | 10.97 | 7.39 | **bio16** | 21.97 | 6.47 | 6.46 | 5.81 | 5.41 |
| **soil_pH** | 8.28 | 8.24 | 7.97 | **pet** | 10.3 | 10.15 | 9.68 | 7.63 | 7.14 |
| **soilC** | 11.15 | 11 | 9.57 | soilC | 11.26 | 11.02 | 10.98 | 10.98 |  |
| **bio16** | 44.83 | 10.39 | 9.68 | annWV | 13.58 | 13.58 | 11.12 |  |  |
| bio15 | 13.69 | 13.06 |  | bio17 | 16.23 | 15.99 |  |  |  |
| ai | 49.28 |  |  | ai | 32.62 |  |  |  |  |
| ***V1* and *V3*** | Run1 |  |  | ***V1* and *V3*** | Run1 |  |  |  |  |
| **bio3** | 2.08 |  |  | **even** | 2.44 |  |  |  |  |
| **bio7** | 2.32 |  |  | **bio8** | 2.67 |  |  |  |  |
| **bio15** | 3.46 |  |  | **bio13** | 2.78 |  |  |  |  |
| **corr** | 3.53 |  |  | **corr** | 3.67 |  |  |  |  |
| **bio13** | 3.64 |  |  | **bio2** | 4.02 |  |  |  |  |
| **max** | 4.06 |  |  | **bio3** | 4.35 |  |  |  |  |
| **cv** | 5.18 |  |  | **bio15** | 5.37 |  |  |  |  |
| **bio19** | 5.56 |  |  | **bio16** | 5.51 |  |  |  |  |
| **even** | 5.59 |  |  | **bio17** | 6.4 |  |  |  |  |
| **bio10** | 5.74 |  |  | **max** | 7 |  |  |  |  |
| **bio17** | 6.17 |  |  | **cv** | 7.16 |  |  |  |  |
| **range** | 7.44 |  |  | **cont** | 8.66 |  |  |  |  |
| **bio16** | 7.66 |  |  | **homo** | 9.91 |  |  |  |  |
| **homo** | 9.83 |  |  |  |  |  |  |  |  |
| ***V1* and *V4*** | Run1 | Run2 |  | ***V1* and *V4*** | Run1 | Run2 |  |  |  |
| **gst** | 1.7 | 1.45 |  | **bio13** | 2.67 | 2.58 |  |  |  |
| **bio7** | 2.12 | 2.04 |  | **bio15** | 7.59 | 5.65 |  |  |  |
| **bio3** | 2.25 | 2.23 |  | **bio16** | 7.01 | 4.61 |  |  |  |
| **gdd** | 3.08 | 3.07 |  | **bio17** | 5.85 | 5.79 |  |  |  |
| **bio15** | 4.38 | 3.41 |  | **bio2** | 5.88 | 4.28 |  |  |  |
| **gsl** | 11.08 | 3.53 |  | **bio3** | 4.75 | 4.36 |  |  |  |
| **bio17** | 6.31 | 4.44 |  | **bio8** | 11.36 | 7.05 |  |  |  |
| **bio16** | 7.12 | 4.45 |  | **gdd** | 1.74 | 1.73 |  |  |  |
| **bio13** | 5.11 | 5.11 |  | **gsl** | 5.50 | 5.38 |  |  |  |
| **bio19** | 5.69 | 5.69 |  | **lgd** | 5.48 | 3.32 |  |  |  |
| bio10 | 11.48 |  |  | gst | 16.64 |  |  |  |  |
| ***V1* and *V5*** | Run1 |  |  | ***V1* and *V5*** | Run1 | Run2 |  |  |  |
| **bio7** | 2.01 |  |  | **bio13** | 2.62 | 2.44 |  |  |  |
| **bio15** | 3.2 |  |  | **bio15** | 7.69 | 5.13 |  |  |  |
| **bio10** | 3.2 |  |  | **bio16** | 5.72 | 5.37 |  |  |  |
| **bio3** | 3.46 |  |  | **bio17** | 7.76 | 5.41 |  |  |  |
| **uvb4** | 4.03 |  |  | **bio2** | 5.86 | 3.78 |  |  |  |
| **bio13** | 4.76 |  |  | **bio3** | 4.37 | 3.47 |  |  |  |
| **uvb2** | 5.02 |  |  | **bio8** | 3.46 | 2.75 |  |  |  |
| **bio17** | 5.21 |  |  | **uvb2** | 7.86 | 4.09 |  |  |  |
| **bio19** | 5.46 |  |  | uvb6 | 10.25 |  |  |  |  |
| **bio16** | 6.41 |  |  |  |  |  |  |  |  |
| ***V1* and *V6*** | Run1 |  |  | ***V1* and *V6*** | Run1 |  |  |  |  |
| **bio3** | 2.22 |  |  | **bio13** | 2.58 |  |  |  |  |
| **bio7** | 2.47 |  |  | **bio8** | 2.85 |  |  |  |  |
| **lulc5** | 2.78 |  |  | **lulc7** | 3.13 |  |  |  |  |
| **lulc7** | 2.8 |  |  | **lulc4** | 3.59 |  |  |  |  |
| **bio15** | 3.65 |  |  | **bio3** | 3.91 |  |  |  |  |
| **lulc4** | 3.68 |  |  | **bio2** | 3.97 |  |  |  |  |
| **lulc10** | 3.76 |  |  | **lulc1** | 4 |  |  |  |  |
| **bio10** | 3.88 |  |  | **lulc6** | 4.06 |  |  |  |  |
| **bio13** | 4.02 |  |  | **bio16** | 4.19 |  |  |  |  |
| **lulc1** | 4.06 |  |  | **bio15** | 4.84 |  |  |  |  |
| **bio19** | 4.83 |  |  | **bio17** | 5.48 |  |  |  |  |
| **lulc6** | 4.89 |  |  |  |  |  |  |  |  |
| **bio17** | 6.26 |  |  |  |  |  |  |  |  |
| **bio16** | 7.68 |  |  |  |  |  |  |  |  |

***V1***, Bioclimatic variables; ***V2***, geo-climatic variables; ***V3***, Habitat heterogeneity; ***V4***, Growing days; ***V5***, Ultra-violet radiations; ***V6***, Consensus land-cover

Refer to Table 1 for the bioclimatic variables.
